# DenseGated-MSNet: A Multi-Sequence Gated Fusion Network for Multiple Sclerosis Classification from Brain MRI

**DOI:** 10.3390/diagnostics16213539

**Published:** 2026-10-28

**Authors:** Suleyman Yaman, Naim Karasekreter, Rabia Çeliköz, Esra Özgül, Hasan Guler

**Affiliations:** 1 Biomedical Department, Vocational School of Technical Sciences, Firat University, Elazig 23119, Türkiye; 2 Department of Computer Engineering, Faculty of Engineering, Afyon Kocatepe University, Afyonkarahisar 03200, Türkiye; karasekreter@aku.edu.tr; 3 Department of Radiology, Afyonkarahisar State Hospital, Afyonkarahisar 03200, Türkiye; rabia.yolalan@saglik.gov.tr; 4 Department of Radiology, Ozgul Clinic, Afyonkarahisar 03200, Türkiye; dresrayam@gmail.com; 5 Electrical-Electronics Engineering Department, Engineering Faculty, Firat University, Elazig 23119, Türkiye; hasanguler@firat.edu.tr

**Keywords:** deep learning, gated feature-level fusion, multiple sclerosis, multi-input classification, multi-sequence brain MRI

## Abstract

**Background/Objectives:** Multiple sclerosis (MS) is a chronic demyelinating disease of the central nervous system, and magnetic resonance imaging (MRI) plays a central role in its diagnosis and monitoring. Because different MRI sequences provide complementary information about MS-related pathology, jointly exploiting multiple sequences may improve automated classification. However, many existing automated MS classification studies rely on limited sequence information, are not consistently evaluated under subject-independent protocols, and are constrained by the scarcity of publicly available multi-sequence datasets organized at the subject level. **Methods:** To address these gaps, we collected and publicly released a new multi-sequence brain MRI dataset and proposed DenseGated-MSNet, a novel end-to-end multi-input deep learning model for MS versus non-MS classification. DenseGated-MSNet processes each co-acquired MRI sequence in parallel through an independent sequence-specific DenseNet-121 branch and integrates the resulting representations through a proposed input-dependent gated feature-level fusion mechanism. Under a strict subject-independent evaluation protocol, the model configuration was determined on a subject-independent development set, and the performance of the final model was internally evaluated using five-fold subject-independent cross-validation on the same cohort at the image and subject levels. **Results:** In internal five-fold subject-independent cross-validation, DenseGated-MSNet achieved a pooled subject-level accuracy of 90.57% (95% CI: 86.44–93.53%) and an AUC of 0.947 (95% CI: 0.918–0.976). The learned gate values also provided sequence-level modulation information, indicating how strongly each MRI sequence was weighted during multi-sequence integration. **Conclusions:** Overall, these findings suggest that DenseGated-MSNet can effectively integrate complementary information from multiple MRI sequences for discriminating established MS from selected non-MS controls, while providing sequence-level modulation information. Together with the released dataset, the proposed framework provides a reproducible basis for future subject-independent MS classification studies.

## 1. Introduction

Multiple sclerosis (MS) is a chronic disease that affects the central nervous system by damaging the protective covering of nerve fibers and disrupting communication between the brain and the rest of the body [1,2,3]. It is one of the leading causes of neurological disability in young and middle-aged adults. MS can lead to a wide range of neurological impairments, including motor dysfunction, sensory deficits, cognitive decline, visual disturbances, and progressive disability, all of which substantially affect quality of life and impose a considerable socioeconomic burden on patients and healthcare systems [1,2,4]. Its global burden has increased markedly in recent decades: the third edition of the Atlas of MS estimated that approximately 2.8 million people were living with the disease worldwide in 2020, corresponding to a global prevalence of about 35.9 per 100,000 population [5], while Global Burden of Disease estimates reported approximately 1.89 million prevalent cases and a prevalence of 23.9 per 100,000 population [6]. These epidemiological findings collectively indicate that the burden of MS has increased over time and that the disease disproportionately affects women [5,6,7]. Although the exact cause of MS remains unclear, current evidence suggests that the disease arises from the interaction between genetic susceptibility and environmental or lifestyle-related risk factors, particularly Epstein–Barr virus infection, vitamin D deficiency, and smoking [1,7,8]. Since disease-modifying therapies are generally more beneficial when initiated early, timely and accurate detection of MS-related pathology is essential for reducing the risk of irreversible neurological damage, guiding treatment selection, and supporting clinical decision-making throughout the disease course [1,3,9]. However, achieving an accurate diagnosis remains challenging because the clinical and radiological presentation of MS is highly heterogeneous and often overlaps with that of other neurological and demyelinating disorders, making disease assessment and differential diagnosis complex tasks that rely heavily on the expertise of experienced clinicians and radiologists [1,10,11,12].

Magnetic resonance imaging (MRI) is the cornerstone imaging modality in the diagnosis and monitoring of MS and is incorporated into current diagnostic criteria and imaging guidelines [9,13,14,15]. Owing to its high sensitivity to focal white-matter lesions and disease-related tissue changes, MRI is routinely used to demonstrate lesion dissemination in space and time, which is essential for the diagnosis of MS [13,15]. Nevertheless, MRI interpretation remains a demanding process that depends on expert visual assessment and may be affected by inter- and intra-reader variability. Misinterpretation of imaging findings has also been recognized as an important contributor to MS misdiagnosis, particularly when MRI abnormalities are evaluated without sufficient clinical context [12,16,17,18]. These challenges become more pronounced during long-term follow-up, as repeated imaging increases the workload for clinicians and radiologists. Accordingly, there is growing interest in automated and reproducible image-analysis tools that can support expert evaluation, improve consistency in image interpretation, and facilitate more efficient clinical decision-making [18,19,20].

A further important consideration is that MS-related abnormalities are not equally visible across all MRI sequences. Different sequences emphasize different tissue contrasts and therefore provide complementary information about lesion burden, lesion distribution, chronic tissue damage, and inflammatory activity. Fluid-attenuated inversion recovery (FLAIR) imaging is particularly useful for detecting MS-related white-matter lesions, whereas T2-weighted and pre- or post-contrast T1-weighted sequences can provide additional information on lesion extent, tissue damage, and active inflammation; acquisition planes may also support anatomical localization and lesion assessment [12,13,15,21]. Therefore, relying on a single MRI sequence may provide only a partial representation of the underlying pathology, whereas integrating multiple sequences may offer a more comprehensive and clinically meaningful basis for automated MS analysis.

Deep learning (DL), particularly convolutional neural networks (CNNs), has become a leading methodology for medical image analysis, learning discriminative representations directly from data and achieving strong results across classification, detection and segmentation tasks [22,23]. In the specific context of MS, DL-based methods have been increasingly applied to MRI data, particularly for lesion detection and segmentation, while several studies have also investigated MRI-based MS classification and reported encouraging performance [18,19,24,25]. These advances point to considerable potential for MRI-based decision-support approaches that could improve the consistency and efficiency of MS evaluation.

Despite this progress, several methodological gaps continue to limit the strength and generalizability of current MRI-based MS classification studies. First, many studies [11,18,26,27,28,29,30,31,32] rely on a single MRI sequence and therefore do not fully exploit the complementary information distributed across sequences. Second, although publicly available multi-sequence MRI datasets exist for MS-related tasks such as lesion segmentation and longitudinal lesion analysis [18,33,34,35], to the best of our knowledge, no publicly available benchmark dataset has been specifically designed for subject-wise MS classification with several co-acquired MRI sequences for each subject. This restricts the development, comparison, and reproducibility of multi-sequence classification frameworks. Third, a substantial proportion of reported approaches [27,28,29,30,32,36,37,38,39,40,41,42] are evaluated using image-level data partitioning that does not strictly separate patients between the training and test sets. In such schemes, slices from the same patient may be present in both subsets, which can inflate performance estimates and weaken claims of generalization [43,44]. Although subject-independent (patient-wise) validation is increasingly recognized as essential for reliable model evaluation [26,31], it is not consistently adopted across the literature for MS classification. Therefore, the high accuracies reported in some MS classification studies should be interpreted with caution.

To address these gaps, we first collected a multi-sequence brain MRI dataset for MS classification. Building on this dataset, we propose DenseGated-MSNet, an end-to-end DL model designed to exploit complementary information from multiple MRI sequences within a unified framework. The proposed model processes each MRI sequence through parallel, sequence-specific branches and extracts discriminative representations independently from each MRI sequence. These features are then integrated through a gated fusion mechanism that adaptively weights the contribution of each sequence to the final decision. The fused representation is then used to produce the final MS versus non-MS prediction.

The main contributions of this study, together with its key methodological novelties, are summarized as follows:

- A new multi-sequence brain MRI dataset for MS versus non-MS classification was collected and made publicly available to support reproducibility and mitigate the limited availability of multi-sequence resources for MS classification.
- A novel end-to-end multi-sequence DL model, DenseGated-MSNet, is proposed to process each MRI sequence through a parallel, sequence-specific branch and integrate the resulting representations using a gated feature-level fusion mechanism.
- The discriminative contribution of individual MRI sequences and their combinations is systematically investigated, providing insight into how complementary sequence-specific information affects MS classification performance.
- A comprehensive experimental analysis is conducted across representative convolutional and transformer-based backbone architectures, and alternative feature-level fusion strategies are systematically compared, including variants with optional post-fusion attention mechanisms.
- The proposed gated fusion mechanism provides sequence-level modulation information, as the learned gates indicate the relative modulation of each MRI sequence within the fused representation.
- A subject-independent evaluation protocol was used in all experiments to avoid data leakage, and the final model was internally evaluated using five-fold subject-independent cross-validation. In addition, the optimistic bias introduced by non-subject-independent image-level partitioning was quantified using the same model and dataset, highlighting the importance of subject-independent evaluation for reliable performance estimation.

The remainder of this paper is organized as follows. Section 2 reviews related studies on automated MS detection and classification. Section 3 presents the methodology, including the newly collected multi-sequence brain MRI dataset, data curation and sequence harmonization procedures, the proposed DenseGated-MSNet architecture, and its gated feature-level fusion mechanism. Section 4 describes the experimental setup and reports the performance of the proposed model together with the systematic ablation studies used to determine its final configuration. Section 5 discusses the main findings in relation to existing MRI-based MS classification studies, examines the sequence-level gate values of the gated fusion mechanism, and outlines the limitations and future directions of the study. Finally, Section 6 concludes the study.

## 2. Related Studies

Automated classification and detection of MS have attracted considerable attention in recent years. The state-of-the-art studies most relevant to the present work are summarized in Table 1. Although several approaches exploit non-imaging signals [36,37,38,41,42], the majority of recent studies [11,26,27,28,29,30,31,32,39,40,45] rely on MRI, reflecting its central role in clinical diagnosis and monitoring of MS. Existing studies employ a wide range of methodological approaches, including compact custom CNNs [11,26,31,37,40], pretrained DL architectures [26,30,42], hybrid DL models [27,36] that combine CNNs with recurrent neural networks (RNNs) and hybrid frameworks [29,30,32] that integrate deep feature extraction (FE) with feature selection (FS) and conventional machine-learning (ML) classifiers. These methodological differences are accompanied by variations in the clinical objectives addressed, ranging from binary MS versus control classification [27,28,32,37,38,39,40,41,42] to MS lesion detection or segmentation [27,31,40], disease activity assessment [26], and differential diagnosis between MS and other neurological [29,36] or demyelinating disorders [11,30].

Within MRI-based studies, FLAIR is the most frequently exploited sequence mainly because of its sensitivity to MS-related white-matter lesions. Therefore, several studies developed single-sequence CNN models using FLAIR images. For example, Yilmaz Acar et al. [31] proposed a custom compact three-layer CNN for distinguishing lesion-bearing from lesion-free FLAIR slices and reported 90.3% accuracy under subject-independent five-fold cross-validation. Shahram et al. [26] evaluated a five-layer custom CNN with pretrained architectures (ResNet50, VGG16, DenseNet-121 and EfficientNetB0), for classifying MS plaques as active or inactive on non-contrast MRI. Their custom model achieved its best performance on FLAIR, with 90.15% accuracy and 94.67% AUC under subject-independent five-fold cross-validation. These findings suggest that custom task-specific models may be competitive with larger pretrained backbones in small MRI datasets, although their generalizability remains dependent on dataset characteristics and validation design.

Another group of studies has focused on hybrid feature-engineering pipelines, in which handcrafted or deep features are followed by FS and conventional ML classifiers. Macin et al. [32], for instance, extracted local phase quantization features from both fixed-size patches and resized whole images, selected the most discriminative features with iterative neighborhood component analysis (NCA), and performed final classification with k-nearest neighbors (KNN). Evaluated with 10-fold cross-validation on axial, sagittal, and combined image sets, the method achieved its best performance on the axial dataset, with 98.37% accuracy. Tatli et al. [30] aggregated deep features from DenseNet201 and ResNet50 through iterative selection and majority voting in a transfer-transfer scheme, achieving 97.63% accuracy for three-class classification of MS, myelitis, and healthy controls on spinal MRI. Similarly, Khattap et al. [39] combined multi-view ResNet50-based feature extraction with attention blocks, quantum-inspired metaheuristic feature selection, and KNN, achieving 98.29% accuracy for MS-versus-healthy classification on brain T2 MRI images. Poyraz et al. [29] also adopted a hybrid strategy based on BrainNeXt-derived deep features, fixed-size image patches, NCA-based feature selection, and SVM classification, improving the accuracy from 94.21% with the standalone BrainNeXt model to 99.73% with the patch-based framework. Although these results are impressive, the multi-stage nature of such pipelines increases the number of tunable components, can complicate reproducibility, and, especially when subject-independent validation is not strictly enforced on small, single-center datasets, may yield optimistic estimates.

Attention mechanisms and transformer architectures constitute another prominent theme. Beyond the attention blocks employed by Khattap et al. [39], Alzahrani [28] proposed MS-Trust, a transformer-based framework that combines a convolutional tokenizer with squeeze-and-excitation and dual causal–global attention. On FLAIR data, MS-Trust achieved 92.85% accuracy for binary MS-versus-healthy classification and 90.79% accuracy for three-class classification of MS, myelitis, and healthy controls under a hold-out validation protocol. Attention has also been used to guide lesion localisation prior to classification; for example, Gopichand et al. [27] coupled attention-assisted segmentation with a recurrent MobileNet-LSTM classifier and reported 95.4% accuracy for distinguishing control and MS images across axial and sagittal views under a hold-out split. Overall, attention-based designs aim to emphasize diagnostically informative regions and channels, but their benefit appears to depend on dataset size and task and is not uniformly observed.

Multi-sequence input remains comparatively less explored in MRI-based MS classification. Most classification-oriented studies rely on a single MRI sequence, despite the complementary diagnostic value of different MRI sequences. To the best of our knowledge, only a limited number of studies have incorporated multiple sequences; however, these sequences have generally been used for sequence-wise comparison [26] or within segmentation-oriented workflows [40] rather than as complementary inputs in an end-to-end classification model. For instance, Shahram et al. [26] acquired T1-, T2-, FLAIR- and contrast-enhanced T1-weighted images for active-versus-inactive MS lesion classification, and evaluated a five-layer custom CNN against pretrained architectures, but the best-performing model was obtained using FLAIR alone. Similarly, Afzal et al. [40] used T1-weighted, T2-weighted, and FLAIR images in a dual patch-wise CNN framework that first segments lesions and then applies a second CNN for MS prediction. Thus, end-to-end MS classification models that explicitly exploit the complementary information of different MRI sequences through sequence-specific representation learning remain largely underexplored. In addition, publicly available multi-sequence MRI datasets are more commonly designed for lesion segmentation or longitudinal lesion analysis than for MS versus control classification [18,33,34,35]. This limits the development, comparison, and reproducibility of multi-sequence classification frameworks.

A further limitation of the existing literature concerns the evaluation protocols used to assess model performance. Many studies report high accuracies under hold-out validation or conventional k-fold cross-validation; however, these protocols do not always clearly ensure subject-level independence between training and test sets. This is particularly important in MRI-based MS classification, where multiple slices, patches, views, or sequences may be obtained from the same subject. If images from the same subject appear in both training and test sets, model performance may reflect subject-specific or acquisition-specific patterns rather than true generalization to unseen patients, creating a risk of patient-level information leakage and inflated accuracy estimates [43,44]. Although subject- or patient-independent evaluation provides a more rigorous assessment of model generalization, it is not consistently adopted across MS classification studies. Some studies explicitly used subject-independent cross-validation [26,31], whereas several others relied on image-level splits or did not provide sufficient detail on subject-level separation. Therefore, rigorous subject-wise evaluation is essential for obtaining reliable and clinically meaningful estimates of model performance.

Beyond MRI, MS detection has also been explored using alternative data sources, including speech recordings [36], surface-enhanced Raman spectroscopy [37], blood-based metabolomics [41], retinal imaging [42], and gait or kinematic analysis [38,46]. These studies illustrate the breadth of data modalities currently being investigated and, in some cases, report high classification accuracies. However, many are based on small sample sizes, modality-specific acquisition protocols, or hold-out evaluation. Since MRI remains the primary modality for assessing MS-related structural pathology [3,13], the present study focuses on MRI-based classification while acknowledging the complementary potential of non-imaging biomarkers.

Overall, the reviewed studies demonstrate substantial progress in automated MS classification and detection using both MRI-based and non-imaging modalities. Most MRI-based classification studies rely on single-sequence inputs, while the complementary diagnostic information provided by different MRI sequences is rarely exploited in end-to-end classification models. In particular, learnable fusion strategies for integrating complementary sequence-specific representations remain underused. Publicly available multi-sequence datasets designed specifically for subject-wise MS classification also remain limited. Moreover, patient-independent evaluation protocols are not consistently adopted, which may lead to optimistic performance estimates and limit the reliability and comparability of reported results. Together, these issues highlight the need for MRI-based MS classification frameworks that better exploit complementary sequence information and are evaluated under rigorous subject-wise validation protocols.

## 3. Methodology

This section presents the methodological framework of the proposed study. First, the general structure and sequence composition of the newly collected multi-sequence brain MRI dataset are introduced. Next, the preprocessing steps used to standardize the MRI images and prepare them for model training are described. Subsequently, the architecture and operational workflow of DenseGated-MSNet are presented in detail. Finally, the gated feature-level fusion mechanism, which represents a key component of the proposed multi-sequence classification framework, is explained.

### 3.1. Dataset and Study Cohort

The dataset used in this study was retrospectively collected from the Radiology Department of Afyonkarahisar State Hospital, Afyonkarahisar, Türkiye, following approval by the Ethics Committee of Afyon Kocatepe University (Afyonkarahisar, Türkiye; 2024/20). The cohort consisted of individuals who underwent brain MRI for neurological evaluation between January 2020 and March 2024. All examinations were acquired on a single 1.5-T MRI scanner (Siemens MAGNETOM Aera, Siemens Healthineers, Erlangen, Germany) using a consistent institutional protocol aligned with European consensus recommendations for MS imaging, including MAGNIMS/ESNR guidance [15,21]. The imaging protocol included FLAIR, T2-weighted, and T1-weighted MRI sequences, acquired in one or more imaging planes depending on the examination. All available FLAIR, T2-weighted, and T1-weighted series were saved from the PACS archive as 2D images in JPEG format, organized by subject and series. For each subject, the available sequences were obtained within the same imaging examination, thereby ensuring that they were co-acquired and matched at the subject level. For subjects with more than one MRI examination during the study period, only the earliest baseline examination was retained, so that each subject was represented by a single examination and appeared only once in the collected dataset.

The cohort included 276 subjects, comprising 134 patients with a clinical diagnosis of MS established according to the revised 2017 McDonald criteria [14] through retrospective review of institutional medical records and PACS archive data, and 142 control subjects who underwent brain MRI for non-MS neurological indications and showed no clinical diagnosis of MS, no radiological evidence of demyelinating white-matter lesions, and no major intracranial abnormalities. These participants are hereafter referred to as non-MS controls.

**Figure 1:**
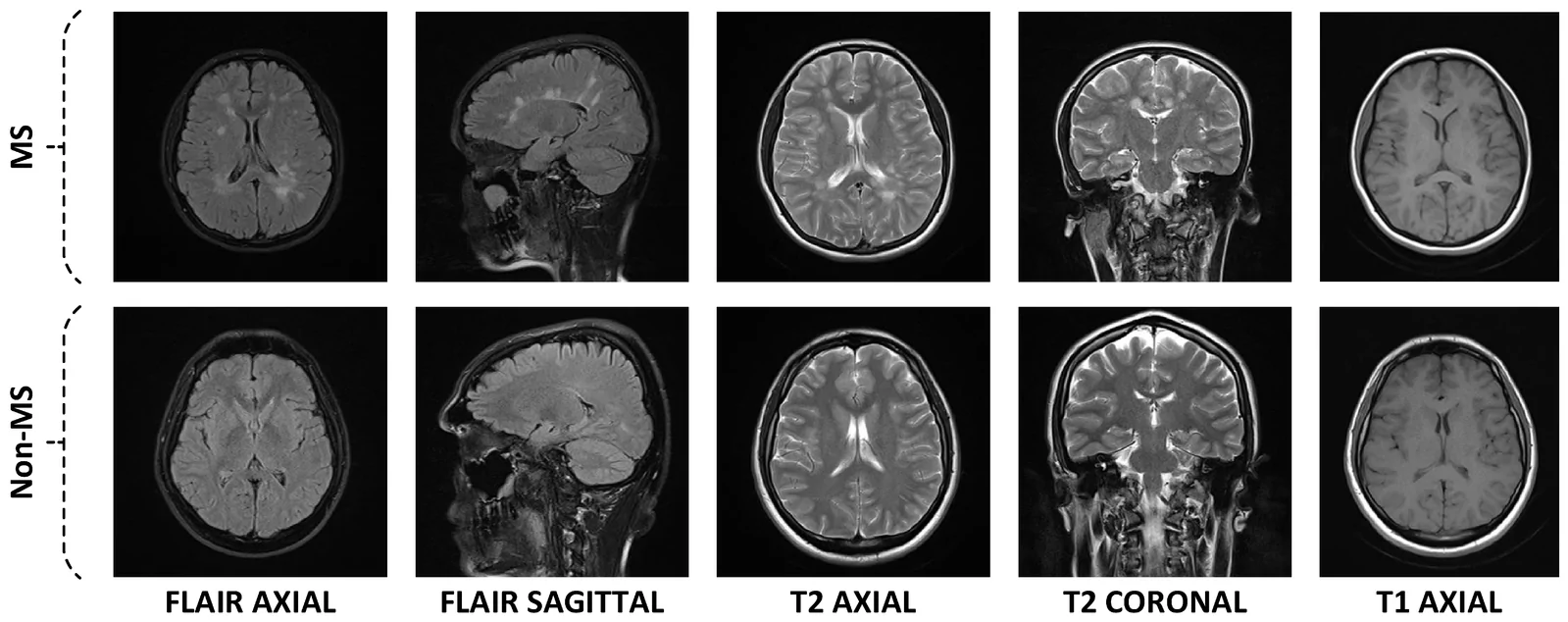
Representative co-acquired MRI slices from the newly collected multi-sequence brain MRI dataset. The upper row shows an MS patient, and the lower row shows a non-MS control across FLAIR axial, FLAIR sagittal, T2 axial, T2 coronal, and T1 axial sequences.

All MRI examinations were independently reviewed by two board-certified radiologists with 8 and 22 years of neuroradiology experience, respectively. The readers were blinded to clinical records and laboratory findings and assessed image quality, sequence-protocol compliance, and imaging findings. Inter-reader agreement for the independent binary classification of MRI examinations based on the presence or absence of demyelinating lesions consistent with MS was 96.4% (266/276 examinations; Cohen’s κ = 0.92). The 10 discordant cases were resolved through joint consensus. Representative co-acquired MRI slices from a non-MS control and an MS patient are shown in Figure 1.

### 3.2. Data Curation and Sequence Harmonization

**Figure 2:**
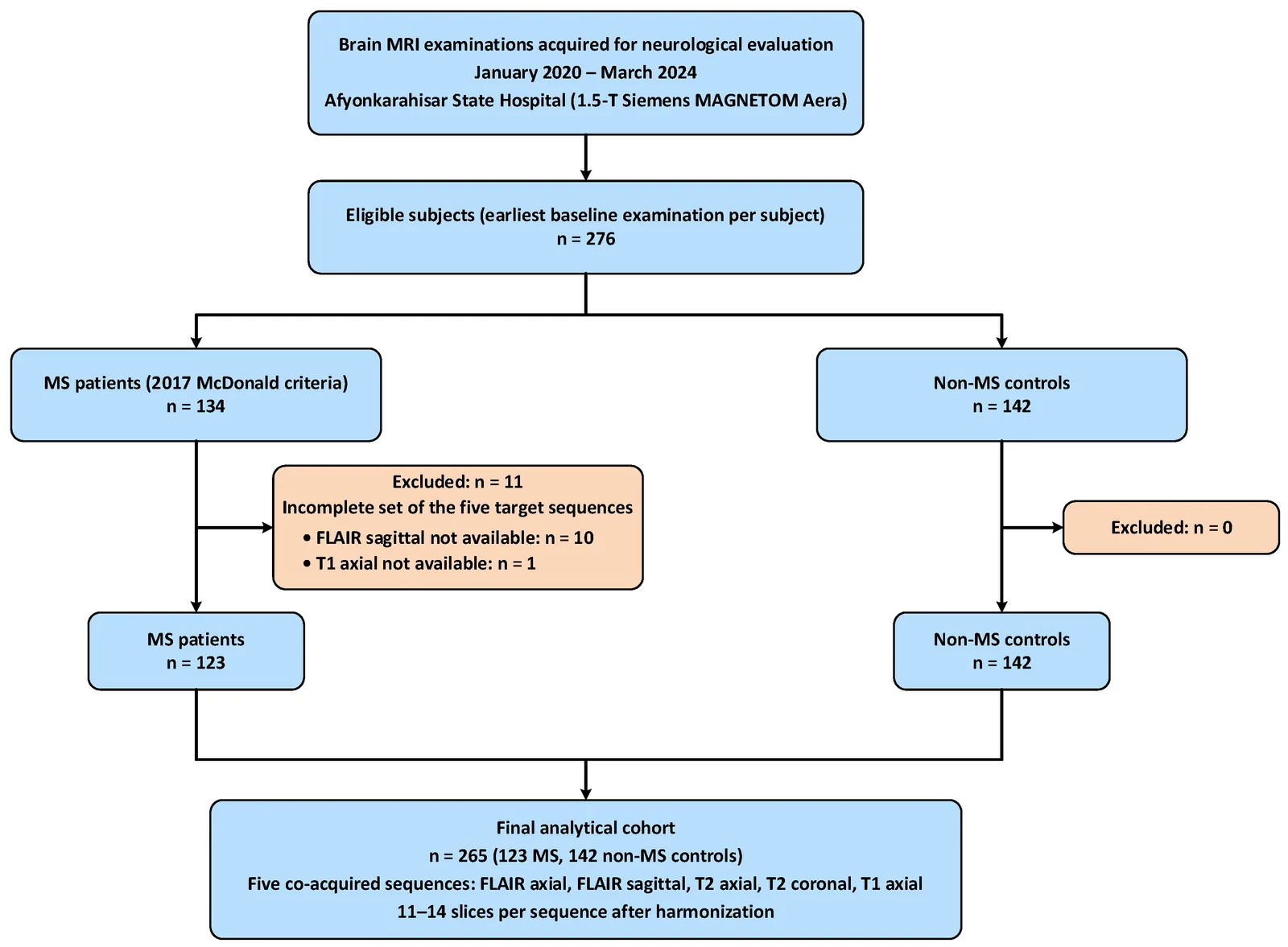
Participant flow diagram of the final analytical cohort.

All MRI examinations were first de-identified at the subject level by removing personal and identifying information. Review of the collected MRI data revealed that the imaging planes available for each sequence type varied across subjects. Accordingly, five sequence–plane combinations were identified as target sequences: FLAIR axial, FLAIR sagittal, T2 axial, T2 coronal, and T1 axial. These five combinations were selected because they represented the largest set commonly available across the cohort, enabling systematic evaluation of individual sequences and their combinations. To ensure that all sequences and sequence combinations were evaluated on the same set of subjects, subjects without the complete set of these five sequences were excluded. This criterion led to the exclusion of 11 MS patients. Of these, FLAIR sagittal images were not available in the retained baseline examinations of 10 patients, three of whom additionally had T2 axial and/or T2 coronal images affected by motion artifacts or poor image quality. In the remaining patient, T1 axial images were not available. All non-MS controls had the complete set of five target sequences with sufficient image quality; therefore, none were excluded. Exclusion was based solely on sequence availability and image quality, rather than on lesion characteristics or clinical findings. The final curated cohort thus comprised 265 subjects, including 123 MS patients and 142 non-MS controls. The participant flow is shown in Figure 2, and the demographic characteristics of the final cohort are summarized in Table 2.

During the initial data inspection, the number of available 2D slices per sequence was found to vary both across MRI sequences within the same subject and across different subjects, ranging from 8 to 30 slices per sequence. For each sequence, the retained slices corresponded to a curated subset selected from the central portion of the acquired volume, where brain parenchyma was more fully represented, while peripheral slices containing little or no brain tissue were excluded. This selection was based on anatomical coverage rather than lesion visibility or diagnostic label, and the same criterion was applied to both MS and non-MS cases. Because the number of retained slices still varied across sequences and subjects, sequence harmonization was performed to obtain a matched multi-sequence structure. For each subject, the largest slice count among the available five sequences was first used as the subject-specific reference. Sequences with fewer slices than this reference were extended to the reference count by duplicating randomly selected slices. This duplication was performed solely to harmonize slice counts across sequences. To limit excessive slice variability across subjects, the maximum reference slice count was capped at 14. Therefore, when a subject had a sequence containing more than 14 slices, all sequences for that subject were harmonized to 14 slices by removing slices symmetrically from the beginning and end of longer sequences and duplicating randomly selected slices where needed. After this procedure, each subject had a matched set of target MRI sequences, with each sequence containing between 11 and 14 slices.

After harmonization, the slices of each sequence were maintained in their acquisition order, and slices with the same index across the five sequences were grouped to form multi-sequence input samples. Thus, each subject contributed N samples, where N denotes the number of harmonized slices per sequence. Since the sequences were acquired in different imaging planes without spatial co-registration, the grouped slices did not necessarily represent the same anatomical location; instead, each sample provides complementary views of the same brain examination. This index-based matching is therefore a pragmatic way of constructing multi-sequence inputs rather than an anatomically validated correspondence. For example, the patient identified as MS10 in the dataset initially had 14, 15, 13, 13, and 10 slices in the FLAIR axial, FLAIR sagittal, T2 axial, T2 coronal, and T1 axial sequences, respectively. To harmonize these sequences, one peripheral slice was removed from FLAIR sagittal, while one, one, and four randomly selected slices were duplicated in T2 axial, T2 coronal, and T1 axial, respectively. Consequently, all five sequences contained 14 slices, yielding 14 multi-sequence input samples for this subject.

Across the final cohort, duplicated slices accounted for 2.2%, 13.5%, and 2.5% of the FLAIR sagittal, FLAIR axial, and T2 coronal images used by the final model, respectively. Since duplicated slices appear multiple times during training and subject-level probability averaging, they may have slightly increased the contribution of the corresponding images. However, random selection ensured that no specific anatomical region was deliberately prioritized. Similarly, truncation to a maximum of 14 slices preserved central brain coverage by removing peripheral slices, although lesions located in the outermost regions may have been underrepresented.

To help address the scarcity of publicly available multi-sequence MRI datasets designed for subject-wise MS versus non-MS classification, and to support reproducibility and fair comparison, this final curated and harmonized dataset is made publicly available at https://kaggle.com/datasets/37bcaead080de3ff5512c905bfea9da0f7d83b4548d0b55d2812695b906efd9a (accessed on 25 October 2026).

### 3.3. Proposed DenseGated-MSNet

In this study, we developed DenseGated-MSNet, an end-to-end DL model for automated MS versus non-MS classification from multi-sequence brain MRI. The model adopts a multi-input architecture that enables several MRI sequences to be processed simultaneously within a unified network. The final architecture described in this section was determined through a systematic series of ablation experiments, including the evaluation of individual MRI sequences, comparison of different backbone families, assessment of multi-sequence learning, and analysis of alternative feature-level fusion strategies with and without optional post-fusion attention mechanisms. The details and results of these ablation studies are presented in the Experiments and Results section.

**Figure 3:**
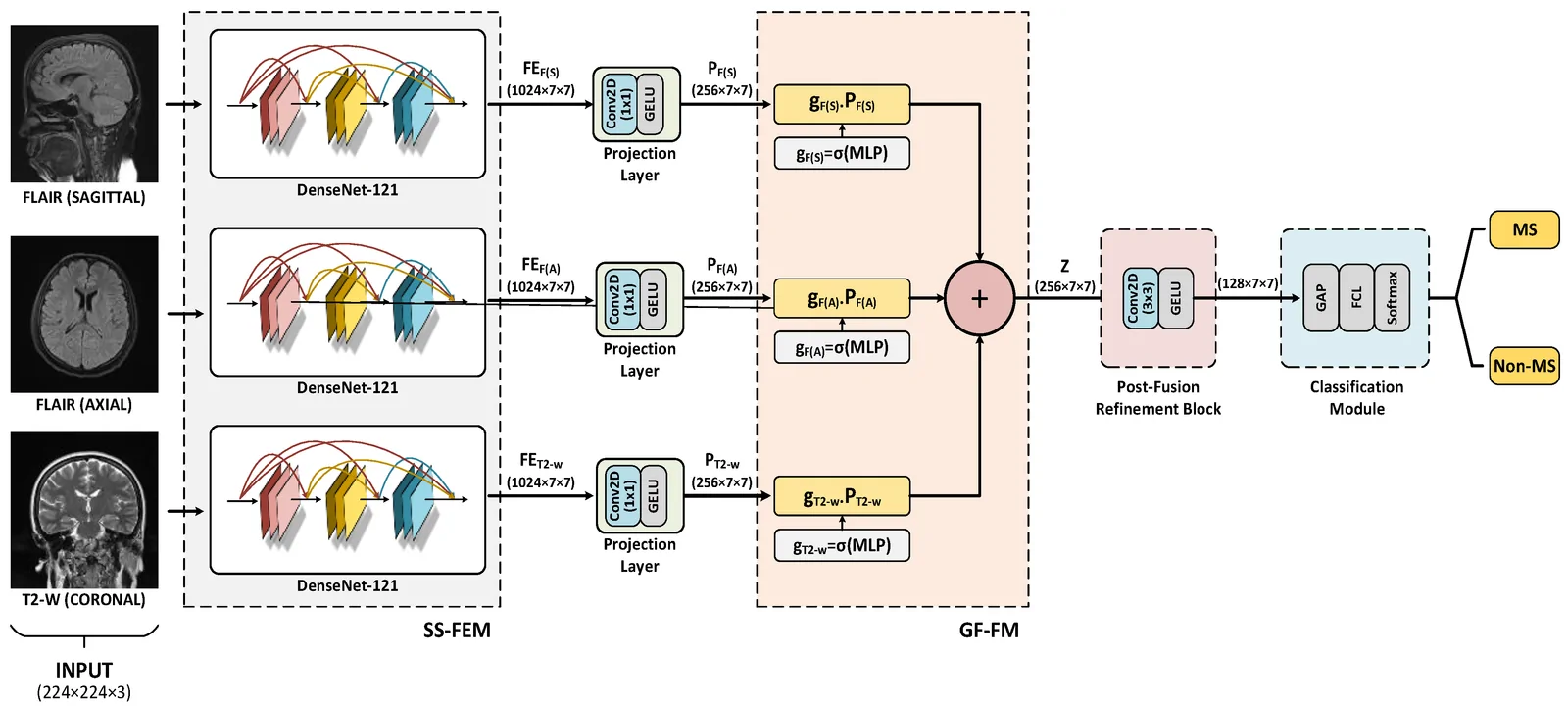
Detailed architecture of the proposed DenseGated-MSNet for multi-sequence MRI-based MS classification.

The proposed DenseGated-MSNet architecture, shown in Figure 3, consists of three main modules: the sequence-specific feature extraction module (SS-FEM), the gated feature-level fusion module (*GF-FM*), and the classification module. This modular design enables the model to first learn informative representations from each MRI sequence separately and then combine these representations in an adaptive manner for the final classification decision. The *SS-FEM* extracts discriminative features from each MRI sequence independently through parallel branches built on DenseNet-121 [47] feature extractors. Since the branches are sequence-specific and do not share parameters, each MRI sequence can be encoded according to its own sequence-specific imaging and pathological characteristics. The resulting feature maps are then passed to the *GF-FM*, where sequence-specific information is integrated into a shared representation. In this module, an input-dependent gate is learned for each sequence, allowing the network to adaptively regulate the relative contribution of each sequence to the fused representation. This design is intended to emphasize complementary and diagnostically relevant information while reducing the influence of less informative sequence-specific features. Finally, the classification module transforms the fused representation into the final MS versus non-MS prediction. In its final configuration, DenseGated-MSNet operates on three MRI sequences, namely FLAIR sagittal, FLAIR axial, and T2 coronal.

DenseGated-MSNet takes as input a set of co-acquired MRI sequence images obtained from the same subject. The model input can be defined as follows:

(1) $$\begin{array}{c} X=\left\{x_{s}\;|\;s=\;1,2,\ldots ,S\right\}\; \end{array}$$

where $x_{s}\in R^{224x224x3}$ represents the image corresponding to the s-th MRI sequence, and *S* denotes the total number of input sequences. In the final configuration of DenseGated-MSNet, S=3, corresponding to FLAIR sagittal, FLAIR axial, and T2 coronal.

The input images are first processed by the *SS-FEM*, which consists of S independent feature extractors operating in parallel, with one branch assigned to each MRI sequence. Each branch employs DenseNet-121 as the backbone encoder. The DenseNet-121 encoders are initialized without ImageNet pretraining and are trained end-to-end as part of the unified DenseGated-MSNet framework. DenseNet-121 was selected for the final architecture based on the systematic backbone comparison conducted in this study, where it achieved the most favorable performance among the evaluated encoder architectures. Since the branches do not share parameters, each MRI sequence is encoded by a dedicated feature extractor, allowing the model to learn sequence-specific representations before fusion. The feature extraction process for the s-th sequence is defined as

(2) $$\begin{array}{c} F_{s}={FE}_{s}\left(x_{s}\right) \end{array}$$

where ${FE}_{s}$ denotes the DenseNet-121 feature extractor corresponding to sequence s, and $F_{s}\in R^{C\;\times \;H\;\times \;W}$ denotes the final convolutional feature map produced by this branch. For an input image of size 224 × 224 × 3, the DenseNet-121 encoder produces a feature map with C=1024 and H=W=7, resulting in $F_{s}\in R^{1024\;\times \;7\;\times \;7}$.

The sequence-specific feature maps extracted by the SS-FEM are then passed to *GF-FM*. Before the gated fusion, each feature map is first processed by a sequence-specific projection layer composed of a 1×1 convolution followed by batch normalization and *GELU* activation [48]. This projection layer maps the encoder output from C channels to a fixed fusion dimension of $C^{\prime }=256$, thereby reducing the computational cost, and it ensures that all sequence-specific representations have a consistent channel dimension before gated fusion, while preserving the spatial resolution of the feature map:

(3) $$\begin{array}{c} P_{s}=GELU\left(BatchNorm\left({Conv}_{1\;\times \;1}^{s}\left(F_{s}\right)\right)\right)\; \end{array}$$

where ${Conv}_{1\;\times \;1}^{s}\left(.\right)$ denotes the sequence-specific *1* × *1* convolution applied to the feature map of sequence s, and $P_{s\;}\in R^{C^{\prime }\times \;H\;\times \;W}$ denotes the projected feature map for sequence *s*. In the final DenseNet-121 configuration, $C^{\prime }=256$ and H=W=7, resulting in $P_{s\;}\in R^{256\;\times \;7\;\times \;7}$.

The projected feature maps $P_{s\;}$ are then adaptively integrated by the gated fusion mechanism to obtain a unified multi-sequence representation. Rather than assigning equal importance to all MRI sequences, the gated fusion mechanism learns an input-dependent gate for each sequence and uses this gate to modulate the corresponding projected feature map, as detailed in Section 3.4. The gated feature maps are first combined by element-wise summation to form an intermediate fused representation:

(4) $$\begin{array}{c} Z=\sum_{s=1}^{S}{\tilde{P}}_{s} \end{array}$$

where ${\tilde{P}}_{s}$ denotes the gated feature map of sequence s, $Z\in R^{C^{\prime }\times H\times W}$ denotes the intermediate fused representation obtained after gated aggregation.

To further refine this fused representation before classification, Z is passed through a post-fusion refinement block, which consists of a 3×3 convolution followed by batch normalization and *GELU* activation:

(5) $$\begin{array}{c} R=GELU\left(BatchNorm\left({Conv}_{3\;\times \;3}\left(Z\right)\right)\right)\; \end{array}$$

where ${Conv}_{3\;\times \;3}\left(.\right)$ denotes 3×3 convolution operation, and $R\in R^{D\times H\times W}$ denotes the refined fused representation produced by *GF-FM*. In the final configuration, D=128 and H=W=7.

The refined fused representation *R* is finally passed to the classification module, where global average pooling is first applied to obtain a compact feature vector. The resulting vector is then processed by fully connected layers with *GELU* activation, followed by a *softmax* function to produce the final MS versus non-MS probabilities:

(6) $$\begin{array}{c} \hat{y}=softmax\left(FCL\left(GAP\left(R\right)\right)\right) \end{array}$$

where FCL(.) denotes the fully connected layers, and $\hat{y}$ represents the predicted probabilities for the non-MS and MS classes.

### 3.4. Gated Feature-Level Fusion and Its Application

Gated feature-level fusion is the mechanism in DenseGated-MSNet responsible for integrating information from different MRI sequences. Because individual MRI sequences differ in their contrast characteristics and sensitivity to MS-related pathology, their contributions to the classification decision are unlikely to be equivalent, and their relative diagnostic relevance may vary across subjects and slices. Therefore, treating all sequences equally or combining them with fixed fusion weights may limit the ability of the model to exploit the most informative sequence-specific features for each input. To address this issue, we developed an input-dependent gated fusion strategy that learns a multiplicative scalar gate for each MRI sequence. This design is inspired by gating-based multimodal fusion approaches, where gates are used to regulate the contribution of different modalities to a shared representation [49,50,51], and by channel-gating attention mechanisms based on global feature descriptors and sigmoid-based recalibration [52]. In the proposed model, we adapt this principle to multi-sequence brain MRI so that DenseGated-MSNet can dynamically emphasize MRI sequences that are more informative for a given sample while attenuating less relevant sequence-specific features. Unlike these approaches, which compute element-wise or spatial gates from joint input representations [49,50,51] or perform channel-wise recalibration of individual feature maps [52], the proposed module independently estimates a single scalar gate for each MRI sequence using its own globally pooled feature descriptor and a sigmoid activation. This design provides an explicit, non-competitive modulation value for each sequence without requiring spatial correspondence for gate estimation. In addition, the learned scalar gates provide a simple sequence-level indication of how strongly each MRI sequence is modulated during fusion, thereby improving the transparency of the fusion process.

The gated fusion mechanism within the GF-FM operates on the projected sequence-specific feature maps $P_{s\;}$, which are obtained from the sequence-specific projection layers. For each sequence, a compact channel descriptor is first obtained by squeezing the spatial dimensions of its projected feature map through global average pooling (*GAP*):

(7) $$\begin{array}{c} d_{s}=GAP\left(P_{s}\right) \end{array}$$

where $d_{s\;}\in R^{C^{\prime }}$ denotes the global descriptor of the s-th sequence. This descriptor summarizes the overall channel response of the projected feature map and provides a compact representation from which the sequence gate is estimated.

The descriptor is then passed through a compact gating network with a bottleneck structure, composed of two *FCL*s with an intermediate *GELU* activation and a final sigmoid activation, which produces a single scalar gate:

(8) $$\begin{array}{c} g_{s}=\sigma \left({FCL}_{2}^{s}\left(GELU\left({FCL}_{1}^{s}\left(d_{s}\right)\right)\right)\right)\; \end{array}$$

where ${FCL}_{1}^{s}\left(.\right)$ and ${FCL}_{2}^{s}\left(.\right)$ denote the sequence-specific FCLs of the gating network, σ denotes the sigmoid function, and $g_{s}\;\in \;\left(0,1\right)$ represents the learned modulation strength of the s-th sequence. In the final configuration, the first *FCL* maps the $C^{\prime }=256$ descriptor to 128 hidden units, and the second *FCL* maps it to a single scalar gate.

The resulting $g_{s}$ is then applied to the corresponding projected feature map through element-wise multiplication:

(9) $$\begin{array}{c} {\tilde{P}}_{s}=g_{s}⊙\;P_{s}\; \end{array}$$

where ⊙ denotes element-wise multiplication with broadcasting, and ${\tilde{P}}_{s}$ represents the gated feature map of the s-th sequence. Since $g_{s}$ is a scalar value, it modulates the overall contribution of the corresponding MRI sequence rather than re-weighting individual channels or spatial locations. This design keeps the fusion mechanism compact while allowing the model to adjust the contribution of each sequence in an input-dependent manner.

Finally, the gated feature maps from all input sequences are aggregated by element-wise summation to form the fused multi-sequence representation, as defined in Equation (4).

Since the input MRI sequences were not spatially co-registered, the element-wise summation of gated feature maps does not imply voxel-wise anatomical correspondence. At this stage, each 7 × 7 feature map is generated by the final dense block of DenseNet-121, whose receptive field encompasses essentially the entire input image. Consequently, each spatial position encodes high-level, image-wide information rather than representing a localized anatomical region. Furthermore, the sequence-specific gates are computed from globally pooled descriptors (Equation (7)), and the fused representation undergoes global average pooling before classification (Equation (6)). Therefore, the proposed fusion mechanism primarily integrates complementary sequence-level information for MS versus non-MS classification rather than combining spatially aligned anatomical regions.

Several design choices distinguish the proposed gated fusion mechanism from alternative feature-level fusion strategies. First, a single scalar gate is learned for each MRI sequence, allowing the model to modulate the overall contribution of that sequence rather than re-weighting individual channels or spatial locations. This keeps the fusion mechanism compact and provides a simple sequence-level indication of how strongly each sequence is modulated during fusion. Second, the gates are estimated from sequence-specific descriptors and generated by independent sigmoid functions rather than a shared softmax normalization. Therefore, the sequences are not forced to compete with each other, and multiple sequences can be simultaneously emphasized or suppressed depending on the input sample, consistent with input-dependent gated multimodal fusion principles. This differs from learnable weighted summation, where trainable weights are used to learn the contribution of different input features [53], and from channel concatenation, which represents a common intermediate fusion strategy but merges all sequences uniformly without explicitly modelling their relative contribution [54]. Because the gates are estimated dynamically from the input features themselves, the sequence modulation can adapt across subjects and slices, which is consistent with the heterogeneous and subject-dependent appearance of MS-related abnormalities across MRI sequences. The comparative performance of these fusion strategies is reported in Section 4.

## 4. Experiments and Results

This section describes the experimental setup, including data partitioning, training and evaluation strategies, and computational resources. The overall performance of the proposed DenseGated-MSNet is then reported, followed by systematic ablation studies that guided the selection of its final configuration. The observed performance trends are also interpreted in relation to the design choices of DenseGated-MSNet.

### 4.1. Experimental Setup

The proposed DenseGated-MSNet was developed through a comprehensive and staged set of ablation studies. Because these ablation studies were extensive and the multi-branch structure of the model increased the computational cost, a subject-independent hold-out split was adopted for the ablation experiments. To prevent data leakage and provide a reliable evaluation, all data partitions were constructed at the subject-independent, also referred to as patient-wise splitting in medical imaging studies, such that all slices belonging to the same subject were kept within a single partition. Accordingly, the curated dataset was divided into a training set of 211 subjects, including 98 MS patients and 113 non-MS controls, and a hold-out evaluation set of 54 subjects, including 25 MS patients and 29 non-MS controls, corresponding approximately to an 80%/20% split. This subject-independent partitioning ensured that neither a subject nor any slice belonging to that subject appeared in both the training and hold-out evaluation sets. Because the hold-out set was used to compare and select model configurations, it served as a development set; in these experiments, the checkpoint achieving the highest accuracy on this set was retained (early stopping patience of 7 epochs), and the hold-out results were therefore not used as the final performance estimate. After the final DenseGated-MSNet configuration had been determined, the model was evaluated using five-fold subject-independent cross-validation. In the cross-validation analysis, subjects were assigned to folds in a class-stratified manner, with all slices from a given subject assigned to the same fold, thereby preventing subject-level data leakage across folds. The subjects of the hold-out evaluation set formed one fold, and the remaining 211 subjects were randomly divided into the other four folds (random seed 42).

All models were trained from scratch, without using pretrained weights, for up to 30 epochs using the AdamW optimizer [55] with a learning rate of 1 × 10^−4^, a weight decay of 1 × 10^−4^, a batch size of 8, and the cross-entropy loss function. No learning-rate scheduling was used. During training, all input slices were transformed online into 224 × 224 × 3 tensors, and normalized channel-wise using a mean and standard deviation of 0.5, mapping intensities to the range [−1, 1]. Light online data augmentation, consisting of random affine transformations with rotations of up to ±5°, translations of up to ±2%, and scaling between 0.98 and 1.02, was applied only to the training partitions to improve robustness and reduce overfitting while preserving the anatomical structure of the MRI slices. No augmentation was applied to the hold-out evaluation set or to the evaluation folds during cross-validation. The main hyperparameters used during training are summarized in Table 3.

In the five-fold cross-validation, the number of training epochs was determined independently for each fold without using the held-out evaluation data. Within each fold, the development subjects were divided in a class-stratified manner into a training subset (approximately 85%) and an internal validation subset (approximately 15%). The model was trained on the training subset for up to 30 epochs, with early stopping based on internal validation accuracy (patience = 10 epochs). The epoch achieving the highest internal validation accuracy was selected. Subsequently, the model was retrained from scratch using all development subjects of that fold for the selected number of epochs. The resulting final-epoch model was evaluated once on the held-out fold, which was excluded from all training and model-selection procedures.

Model performance was assessed using metrics appropriate to each experimental stage. For the ablation studies, model configurations were compared at the image level using accuracy and F1-score. Accuracy was used to assess overall classification performance, whereas F1-score was included because of the mildly imbalanced class distribution. After the final DenseGated-MSNet configuration had been selected, a comprehensive evaluation was performed using standard classification metrics [56], including accuracy (Acc), sensitivity/recall (Sen), specificity (Spe), precision (Pre), F1-score, and the area under the receiver operating characteristic curve (AUC), with MS considered as the positive class. For the final model, metrics were computed at both image and subject levels. Image-level metrics were calculated based on individual MRI images, whereas subject-level metrics were obtained by averaging the predicted MS probabilities across all images belonging to the same subject and applying a decision threshold of 0.5. For the subject-level cross-validation results, 95% confidence intervals (CIs) were computed from the predictions pooled across the five folds, using the Wilson method [57] for accuracy, sensitivity, specificity, and precision, DeLong’s method [58] for the AUC, and stratified bootstrap resampling (2000 iterations) [59] for the F1-score. These pooled out-of-fold estimates, computed once from all 265 subject-level predictions, were considered the primary subject-level performance estimates; fold-wise mean ± standard deviation values are additionally reported to describe variability across folds.

All experiments were implemented in Python (version 3.11) using the PyTorch DL framework (version 2.12.1) [60], with backbone architectures instantiated through the timm library (version 1.0.28). Model training and evaluation were performed on a workstation equipped with an NVIDIA GeForce RTX 4090 GPU with 24 GB of memory and 128 GB of system RAM.

### 4.2. Performance of the Proposed DenseGated-MSNet

In this study, the final DenseGated-MSNet configuration was determined through a comprehensive series of systematic ablation studies investigating the effects of backbone architecture, individual MRI sequences and their combinations, feature-level fusion strategy, and post-fusion attention on classification performance. Based on these analyses, the proposed model adopted DenseNet-121 as the sequence-specific backbone, FLAIR sagittal, FLAIR axial, and T2 coronal as the three input sequences, gated feature-level fusion as the fusion mechanism, and no additional post-fusion attention module. A detailed description of all ablation studies and their results is provided in Section 4.3.

The performance of the proposed DenseGated-MSNet was evaluated using five-fold subject-independent cross-validation; for completeness, the results on the hold-out set, averaged over three random seeds (42, 43, and 44), are also reported. In both protocols, all images belonging to the same subject were kept within a single partition or fold, thereby preventing subject-level data leakage. The results are summarized in Table 4.

The hold-out evaluation demonstrated that the proposed DenseGated-MSNet achieved high classification performance at both image and subject levels. At the image level, the model reached 96.05 ± 0.95% accuracy and 95.65 ± 1.02% F1-score, indicating strong discriminative capability for individual MRI images. When predictions were aggregated at the subject level, performance further increased to 97.53 ± 1.07% accuracy and 97.33 ± 1.15% F1-score, with image- and subject-level AUCs of 0.9929 ± 0.0032 and 0.9986 ± 0.0014, respectively. This improvement indicates that aggregating the image-level probabilities into a single subject-level decision reduces the influence of individual ambiguous images and yields a more stable prediction, which is also the clinically more meaningful level of evaluation.

A similar trend was observed in the five-fold subject-independent cross-validation, although, as expected, the overall performance was lower than that obtained in the hold-out setting, which was used for model selection. At the image level, DenseGated-MSNet achieved 88.93 ± 2.31% accuracy and 87.84 ± 2.42% F1-score, while subject-level aggregation again increased the performance, with a pooled accuracy of 90.57% (95% CI: 86.44–93.53%) and F1-score of 89.80% (85.71–93.44%) (fold-wise mean: 90.54 ± 4.69% and 89.87 ± 4.77%). Sensitivity and specificity were relatively balanced, with values of 87.32% and 90.30% at the image level and 89.43% and 91.55% at the subject level (pooled), respectively. This is reflected in the confusion matrices aggregated over the five test folds, where 130 of 142 non-MS controls and 110 of 123 MS subjects were correctly classified at the subject level, while 1792 of 1985 non-MS images and 1461 of 1673 MS images were correctly classified at the image level. Overall, the five-fold cross-validation results provide a more conservative internal estimate of the model’s performance under subject-independent evaluation.

**Figure 4:**
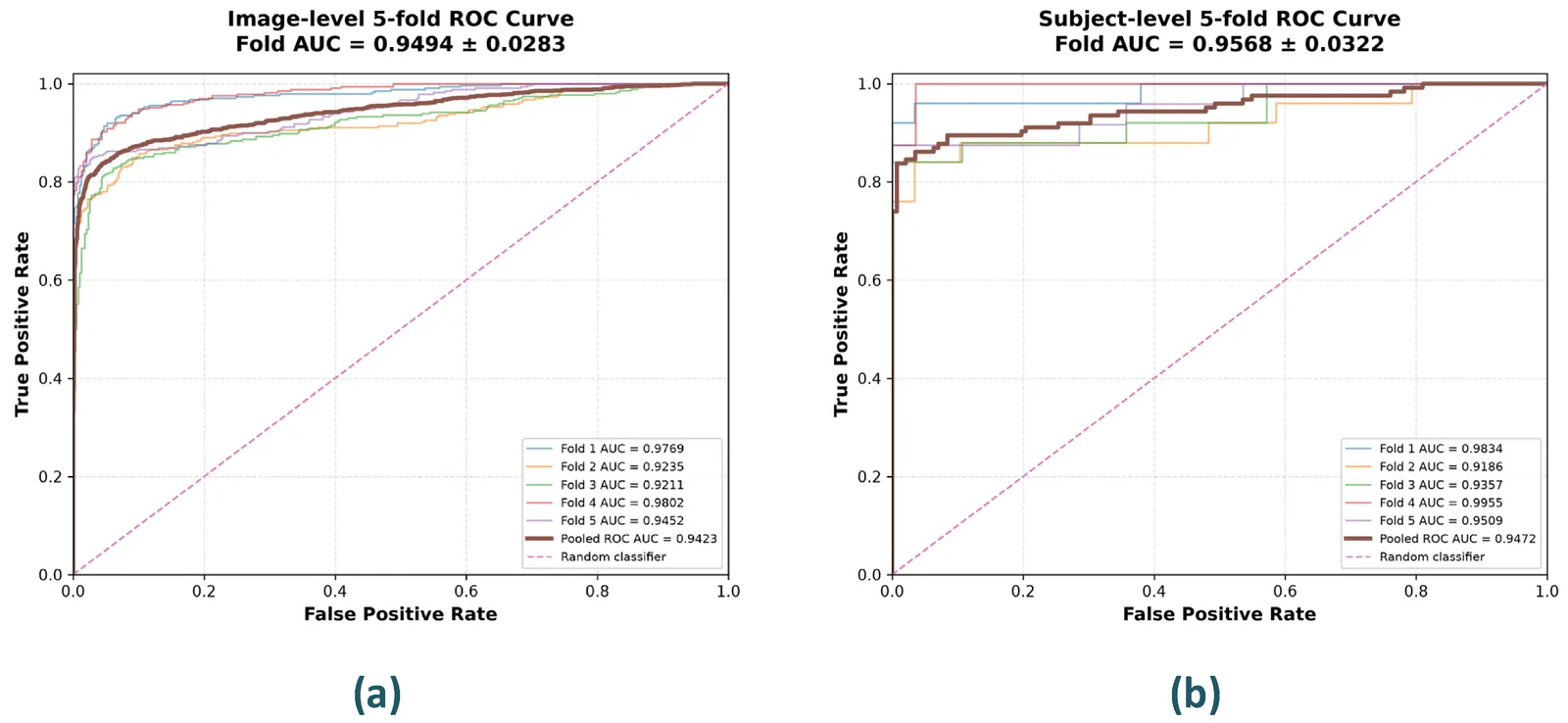
ROC curves of DenseGated-MSNet across the five subject-independent cross-validation folds, together with the pooled ROC: (**a**) image level and (**b**) subject level.

The discriminative ability of DenseGated-MSNet under five-fold subject-independent cross-validation is further illustrated by the image-level and subject-level ROC curves in Figure 4. Each plot includes the ROC curves of the five individual folds together with the corresponding pooled ROC curve. The pooled AUCs were 0.942 at the image level and 0.947 (95% CI: 0.918–0.976) at the subject level, compared with mean fold-wise AUCs of 0.949 ± 0.028 and 0.957 ± 0.032, respectively. While the mean fold-wise AUC represents the average of the AUCs calculated separately for each fold, the pooled AUC is calculated from the combined out-of-fold predictions of all five fold-specific models. Therefore, the pooled AUC may be slightly lower because differences in probability calibration between fold-specific models can affect the ranking of predictions when combined. The individual fold ROC curves showed broadly consistent patterns. This indicates that the discriminative performance of the model was stable across different subject-independent partitions rather than being driven by a single fold. Overall, these results further support the ability of DenseGated-MSNet to provide reliable discrimination between MS and non-MS cases under a strict subject-independent evaluation protocol.

To assess the potential impact of reusing the hold-out evaluation set for model configuration selection in the ablation studies (Section 4.3), an additional analysis was performed, as these subjects also constituted one of the five cross-validation folds. When performance was calculated using only the remaining four folds (211 subjects), excluding the fold previously used for configuration selection, the pooled subject-level accuracy was 89.10% (95% CI: 84.17–92.63%), with a sensitivity of 87.76% (79.81–92.85%), a specificity of 90.27% (83.41–94.48%), and an AUC of 0.944 (0.912–0.977). These values were close to those obtained across all five folds (accuracy 90.57%, AUC 0.947). Although this finding suggests that the reuse of hold-out subjects did not substantially inflate the cross-validation performance estimates, it does not fully eliminate the potential dependence between model configuration selection and performance estimation.

As described in Section 3.2, the slice-harmonization procedure introduced duplicated slices. To assess their potential influence on cross-validation performance, a sensitivity analysis was conducted by excluding all multi-sequence input samples containing at least one duplicated slice from the evaluation, without retraining the models (636 of 3658 samples, 17.4%). Performance was then recalculated using the remaining 3022 samples, with at least seven samples retained per subject. At the subject level, accuracy, sensitivity, specificity, precision, and F1-score remained unchanged from the values reported in Table 4, whereas the pooled AUC changed marginally from 0.947 to 0.948. Moreover, none of the 265 subjects changed their predicted class. At the image level, accuracy, sensitivity, specificity, F1-score, and AUC were 89.46 ± 2.34%, 88.33 ± 4.69%, 90.27 ± 4.30%, 87.90 ± 2.76%, and 0.953 ± 0.028, respectively, with differences of at most approximately 1 percentage point compared with the results obtained using all samples. These findings indicate that excluding duplicated samples at evaluation had little effect on the reported predictions. However, because duplicated samples were still included during model training, this analysis does not establish that duplication had no influence on model development.

### 4.3. Systematic Ablation Studies

In this study, a comprehensive set of ablation experiments was conducted to address the main research questions and to experimentally examine the methodological assumptions underlying the proposed framework. Specifically, these analyses progressively evaluated the performance of representative DL backbones for MS versus non-MS classification together with the discriminative contribution of individual MRI sequences, the complementary effect of integrating multiple MRI sequences, the influence of alternative feature-level fusion strategies, and the role of post-fusion attention-based refinement. The findings obtained from these experiments were subsequently used to guide and substantiate the final DenseGated-MSNet architecture. All ablation experiments were performed under the subject-independent hold-out setting.

#### 4.3.1. Backbone Benchmarking and Sequence-Wise Performance Analysis

**Figure 5:**
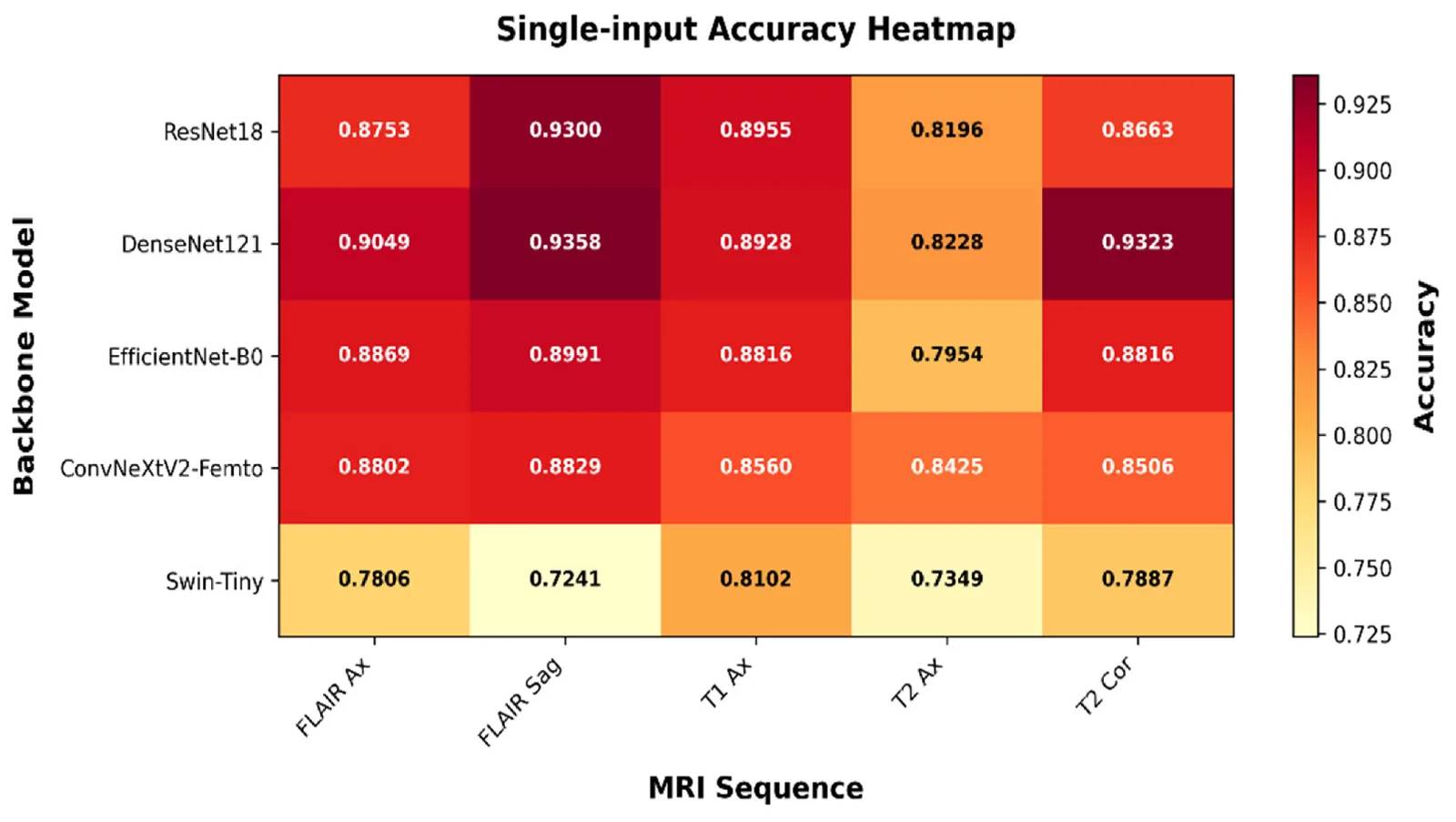
Single-input image-level accuracy per backbone and MRI sequence.

**Figure 6:**
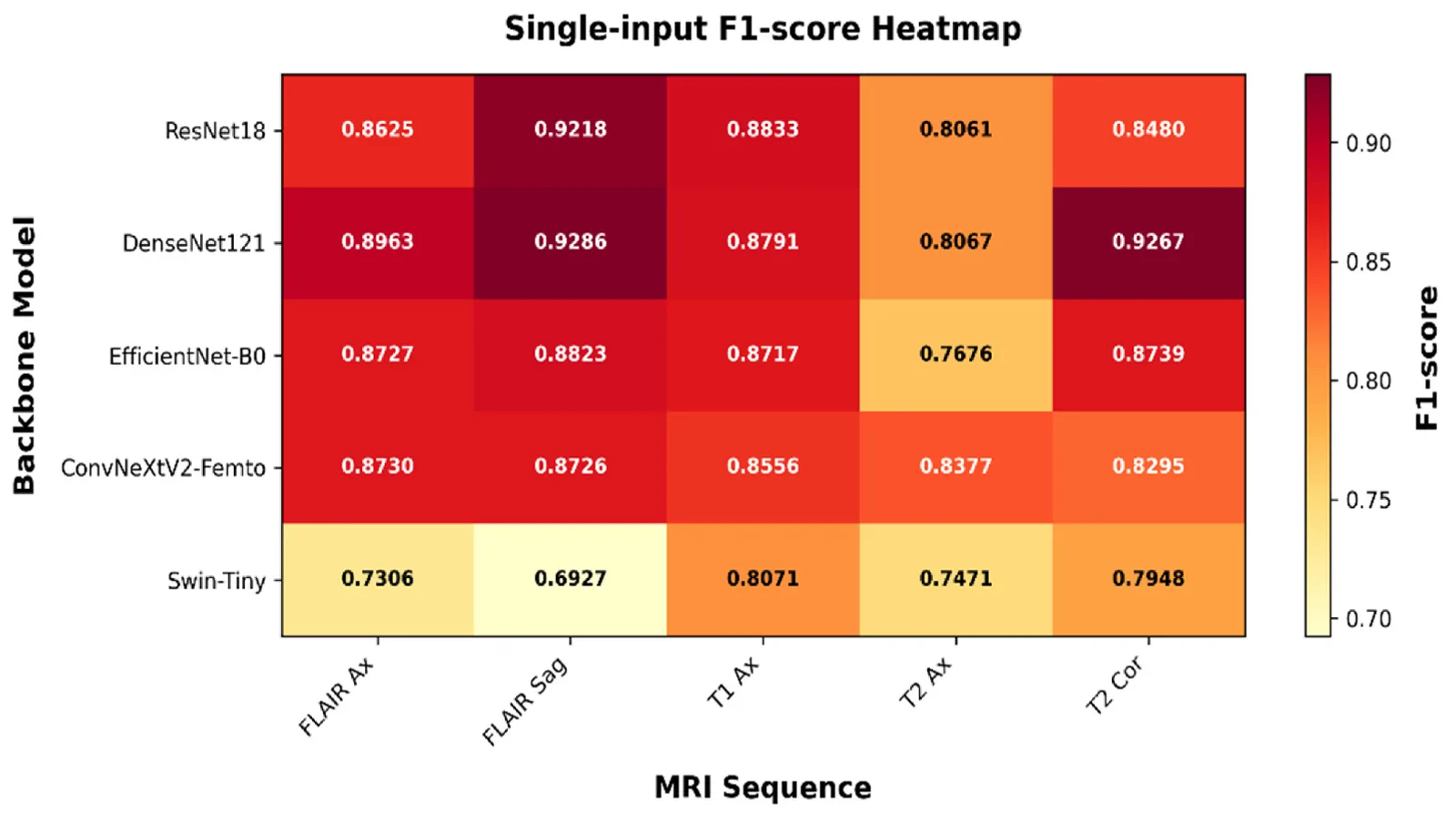
Single-input image-level F1-score per backbone and MRI sequence.

The first ablation study was designed to jointly address two key questions: which backbone architecture is most suitable for MS versus non-MS classification, and which MRI sequences provide the strongest individual discriminative information. For this purpose, five representative DL backbones, namely ResNet18 [61], DenseNet-121 [47], EfficientNet-B0 [62], ConvNeXtV2-Femto [63], and Swin-Tiny [64], were independently trained as single-input classifiers on each of the five MRI sequences: FLAIR axial, FLAIR sagittal, T1 axial, T2 axial, and T2 coronal. This resulted in 25 single-sequence models. In this single-input setting, no fusion module was used, and all models were trained with the same classification head and training protocol to ensure a fair comparison. Each model was trained and evaluated independently under the subject-independent hold-out setting, and image-level accuracy and F1-score were computed on the hold-out evaluation set. The resulting accuracy and F1-score matrices are presented as heatmaps in Figure 5 and Figure 6, respectively.

At the backbone level, DenseNet-121 demonstrated the strongest and most consistent performance among the evaluated architectures. It achieved the highest accuracy on three of the five sequences, namely FLAIR axial, FLAIR sagittal, and T2 coronal, with corresponding accuracies of 90.49%, 93.58%, and 93.23%, respectively. It also produced the best overall single-sequence performance among all single-input experiments, reaching 93.58% accuracy and 92.86% F1-score on FLAIR sagittal. This superiority was further supported by its mean performance across all five MRI sequences, where DenseNet-121 achieved the highest average accuracy and F1-score, with values of 89.77% and 88.75%, respectively. ResNet18 and EfficientNet-B0 also showed competitive performance, with ResNet18 achieving the highest accuracy on T1 axial at 89.55%. However, their average performances remained lower than that of DenseNet-121, with ResNet18 reaching a mean accuracy of 87.73% and a mean F1-score of 86.43%, while EfficientNet-B0 achieved 86.89% and 85.36%, respectively. ConvNeXtV2-Femto yielded slightly lower overall performance than DenseNet-121 but showed relatively robust performance on T2 axial; its average accuracy and F1-score across sequences were 86.24% and 85.37%, respectively. In contrast, Swin-Tiny performed substantially worse than the convolutional backbones across most sequences, with accuracies ranging from 72.41% to 81.02% and the lowest average accuracy and F1-score across sequences, corresponding to 76.77% and 75.45%, respectively. These results suggest that the available dataset may not have been sufficient to effectively train a transformer-based architecture from scratch, whereas convolutional backbones were better suited to the limited-data setting of this study.

At the sequence level, the heatmaps revealed clear differences in the discriminative value of individual MRI sequences. When averaged across all backbones, FLAIR sagittal achieved the highest overall accuracy and F1-score, with values of 87.44% and 85.96%, respectively, indicating that it was the most consistently informative individual sequence. T1 axial also showed competitive average performance, achieving an accuracy of 86.72% and an F1-score of 85.94%. FLAIR axial and T2 coronal provided similarly strong average performance, with accuracies of 86.56% and 86.39% and F1-scores of 84.70% and 85.46%, respectively. In contrast, T2 axial showed the lowest average performance overall, with a mean accuracy of 80.30% and a mean F1-score of 79.30%, suggesting that it provided the weakest single-sequence discriminative information in this experimental setting, although ConvNeXtV2-Femto performed comparatively well on this sequence. The F1-score heatmap generally mirrored the accuracy trends, indicating that the observed performance differences were not merely an artifact of the accuracy metric. Nevertheless, sequence selection for the subsequent multi-sequence experiments was based on the performance of the selected backbone. For DenseNet-121 specifically, FLAIR sagittal and T2 coronal were the two most informative sequences, while FLAIR axial also provided strong classification performance. T1 axial, despite its competitive average performance across backbones, ranked fourth for DenseNet-121. These findings indicate that the sequences differ substantially in their diagnostic value for automated MS detection, which is in line with the radiological understanding that FLAIR-based sequences and different acquisition planes emphasize MS-related white-matter changes to differing degrees.

Based on these results, DenseNet-121 was selected as the backbone architecture for the subsequent multi-sequence experiments. In addition, the three most discriminative sequences for DenseNet-121, namely FLAIR sagittal, FLAIR axial, and T2 coronal were carried forward to the multi-sequence integration experiments. Although T1 axial showed competitive average performance across backbones, it was not retained because it ranked below these three sequences for the selected backbone. T2 axial was excluded because it showed the weakest overall performance among the evaluated sequences.

#### 4.3.2. Effect of Multi-Sequence MRI Integration

**Figure 7:**
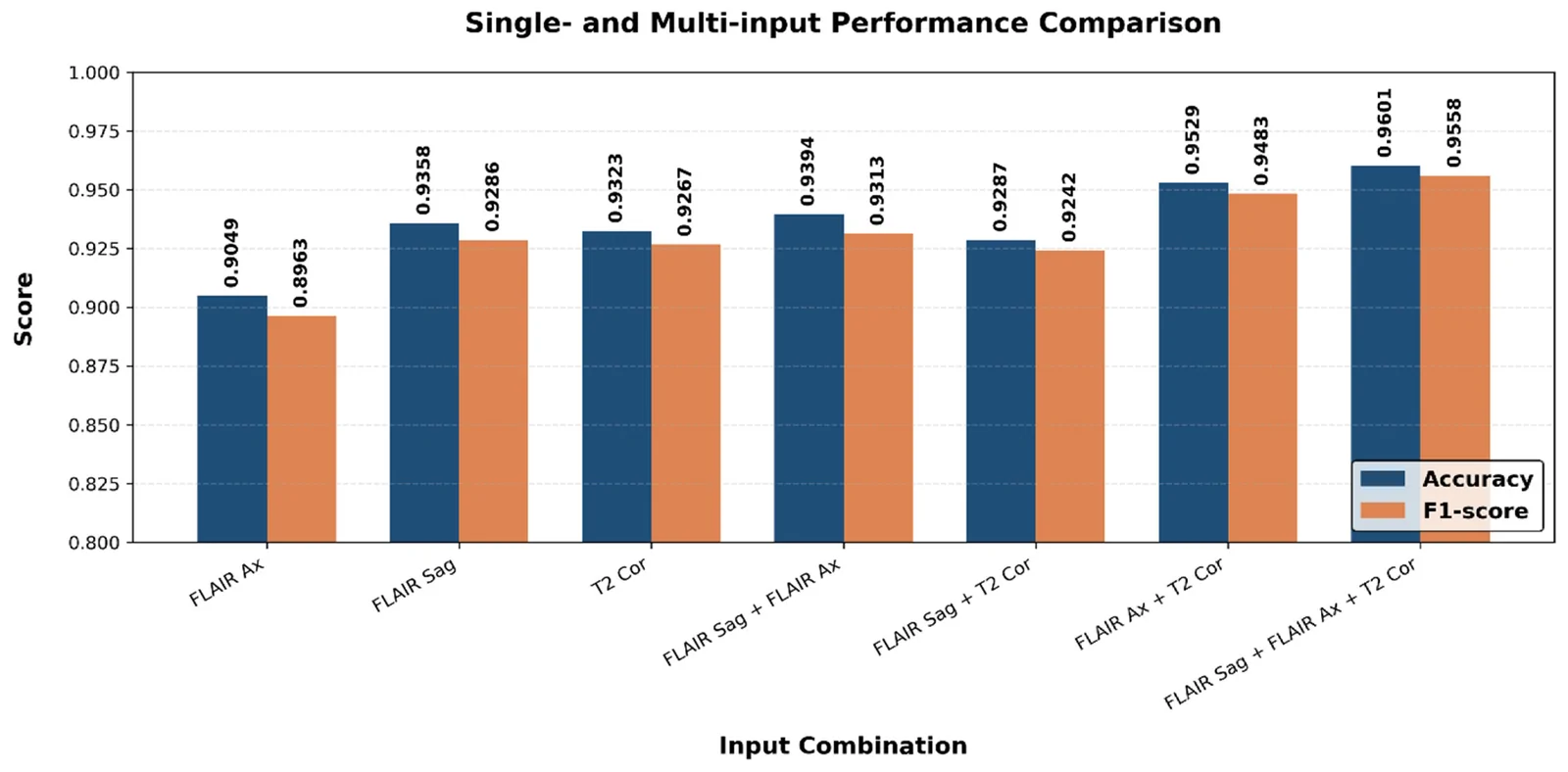
Image-level accuracy and F1-score of single- and multi-sequence DenseNet-121 models for different input combinations.

One of the main research questions of this study is whether jointly exploiting complementary information from multiple MRI sequences can improve MS versus non-MS classification compared with relying on individual sequences alone. To address this question, the second ablation study evaluated the effect of multi-sequence MRI integration using DenseNet-121 as the fixed backbone. Based on the findings of the previous ablation study, the three most discriminative sequences for DenseNet-121, namely FLAIR sagittal, FLAIR axial, and T2 coronal, were evaluated in all pairwise combinations and as a three-sequence configuration. The resulting multi-input models were then compared with their corresponding single-sequence baselines. Each multi-input model was implemented and trained as a single end-to-end network, rather than as a combination of separately trained sequence-specific models. In this architecture, each MRI sequence was processed by an independent DenseNet-121 branch with non-shared parameters, and the resulting sequence-specific feature maps were integrated using channel-concatenation fusion. Specifically, the extracted feature maps were concatenated along the channel dimension and transformed into a unified fused representation before classification. The classification head and training protocol were kept consistent with the single-input experiments, and all models were evaluated under the subject-independent hold-out setting at the image level. The image-level accuracy and F1-score values for the single sequences and their combinations are shown in Figure 7.

The results showed that multi-sequence integration generally improved classification performance, although the magnitude of improvement varied depending on the selected sequence combination. The three-sequence configuration composed of FLAIR sagittal, FLAIR axial, and T2 coronal achieved the best overall performance, reaching 96.01% accuracy and 95.58% F1-score. This result outperformed all single-sequence and pairwise configurations, indicating that the joint use of complementary MRI sequences provided a more discriminative representation than any individual sequence alone. Among the pairwise models, the combination of FLAIR axial and T2 coronal yielded the strongest performance, with 95.29% accuracy and 94.83% F1-score, exceeding the best single-sequence baseline obtained with FLAIR sagittal alone. Notably, although FLAIR axial was the weakest of the three selected sequences when used individually, its combination with T2 coronal produced a marked performance gain, suggesting that these two sequences captured complementary information relevant to MS classification. In contrast, not all sequence combinations were equally beneficial. The FLAIR sagittal + FLAIR axial configuration provided only a marginal improvement over the best single-sequence model, achieving 93.94% accuracy and 93.13% F1-score, whereas the FLAIR sagittal + T2 coronal combination achieved 92.87% accuracy and 92.42% F1-score, performing slightly below its corresponding constituent single-sequence models.

Overall, these findings indicate that multi-sequence MRI integration can improve MS versus non-MS classification, but the observed gain depends on the complementary relationship between the selected sequences rather than on the number of inputs alone. The superior performance of the three-sequence configuration supports the central premise of this study that jointly exploiting complementary information from FLAIR sagittal, FLAIR axial, and T2 coronal images yields a more discriminative representation than relying on single-sequence inputs. Importantly, this configuration also combines information from sagittal, axial, and coronal acquisition planes, suggesting that complementary anatomical perspectives, together with sequence-specific contrast information, may contribute to improved MS classification performance. However, this does not appear to be governed by plane or contrast diversity alone, as the FLAIR sagittal + T2 coronal combination, which also spans different planes and contrasts, did not yield such a gain; this indicates that sequence complementarity is empirical rather than fully predictable from acquisition geometry. Based on these results, this three-sequence configuration was adopted as the input configuration for the subsequent experiments.

#### 4.3.3. Effect of Feature-Level Fusion Strategies

In multi-input DL models, the fusion strategy used to integrate representations extracted from different inputs is a critical design choice that can substantially influence classification performance. Different fusion mechanisms impose different assumptions on how sequence-specific features interact, how complementary information is preserved, and how the relative contribution of each input is represented during decision-making. Therefore, fusion strategy constitutes a central methodological component of a multi-sequence classification framework. Accordingly, this ablation study assessed the effect of different feature-level fusion strategies on model performance. For this purpose, three fusion strategies, namely channel concatenation, learnable weighted summation, and the proposed gated fusion, were compared within the same end-to-end DenseNet121-based architecture using the established three-sequence input configuration.

**Figure 8:**
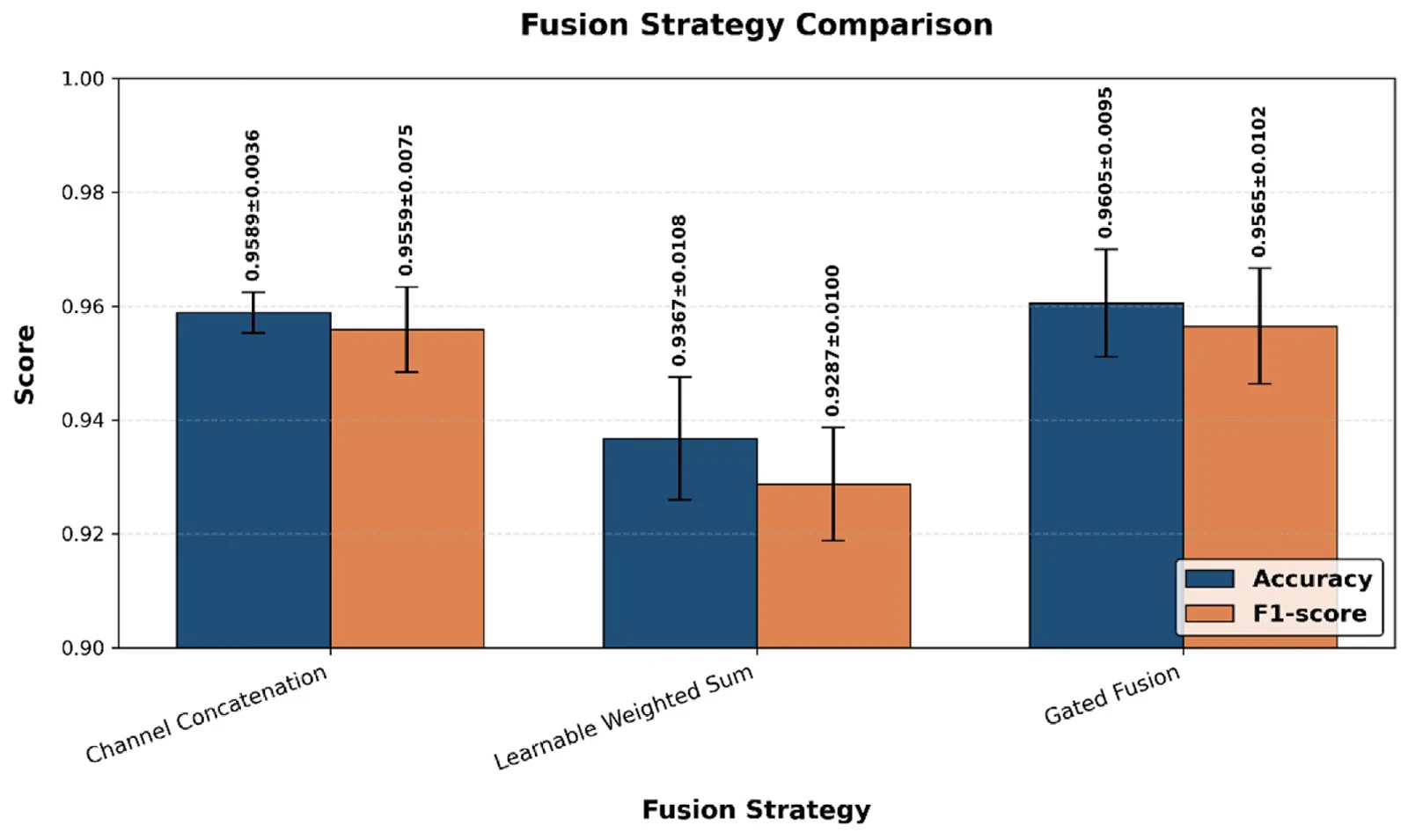
Image-level accuracy and F1-score of the feature-level fusion strategies (mean ± standard deviation over three seeds).

The three strategies differ only in how the sequence-specific feature maps are combined into the fused representation; the subsequent post-fusion refinement and classification stages are identical across all of them. In the channel-concatenation strategy, the branch feature maps are concatenated along the channel dimension, and a 1 × 1 convolution is then applied to reduce the resulting high-dimensional tensor to the common fusion dimension of 256 while enabling feature mixing across sequences and channels. In this strategy, the sequences are therefore combined uniformly, and their relative importance is learned only implicitly through the convolutional weights. In learnable weighted summation, each feature map is first projected to a common channel dimension of 256, and the projected maps are then combined using a single learnable weight per sequence. These weights are normalized by a softmax and shared across all inputs. Therefore, the same fixed weighting scheme is applied to every sample, making the fusion process input-independent and forcing the sequences to compete with one another. In contrast, the proposed gated fusion strategy modulates each sequence using an input-dependent scalar gate before aggregation, allowing the relative contribution of each sequence to be adjusted on a per-sample basis without softmax-based competition, as described in Section 3.4. All three strategies were integrated into the fusion stage of the same model architecture and trained under identical conditions. To account for variability arising from random initialization, each configuration was trained using three random seeds, namely 42, 43, and 44. The image-level accuracy and F1-score values on the hold-out evaluation set are reported as mean ± standard deviation in Figure 8.

The results revealed clear differences in performance among the three fusion strategies. Gated fusion achieved the highest mean performance, reaching 96.05 ± 0.95% accuracy and 95.65 ± 1.02% F1-score. Channel concatenation performed very similarly, obtaining 95.89 ± 0.36% accuracy and 95.59 ± 0.75% F1-score, whereas learnable weighted summation showed lower performance, achieving 93.67 ± 1.08% accuracy and 92.87 ± 1.00% F1-score. These results indicate that both gated fusion and channel concatenation were more effective than fixed learnable weighting for integrating complementary sequence-specific features. The weaker performance of learnable weighted summation may be related to its reliance on a single weighting scheme shared across all samples, which prevents the model from adapting the relative contribution of each sequence to the specific input. In addition, the softmax normalization forces the sequences to compete with one another. In contrast, channel concatenation allows joint feature mixing through convolutional transformations, while gated fusion provides adaptive sequence-level modulation on a per-sample basis.

Although gated fusion attained the highest mean accuracy and F1-score, its advantage over channel concatenation was small, and the standard-deviation ranges of the two strategies overlapped. Therefore, these two strategies can be regarded as broadly comparable in terms of overall performance on this dataset, with channel concatenation exhibiting lower variability across random seeds. Nevertheless, gated fusion was adopted as the fusion mechanism of the final DenseGated-MSNet model for two main reasons. First, it achieved the best mean performance among the evaluated strategies. Second, unlike channel concatenation, which fuses all sequences uniformly, gated fusion explicitly models the contribution of each sequence in an input-dependent manner. This provides a more explicit and adaptive integration mechanism that is conceptually aligned with the heterogeneous and subject-dependent appearance of MS-related abnormalities across MRI sequences. Based on these findings, gated fusion was selected as the feature-level fusion strategy for the subsequent experiments.

#### 4.3.4. Effect of Post-Fusion Attention-Based Refinement

**Figure 9:**
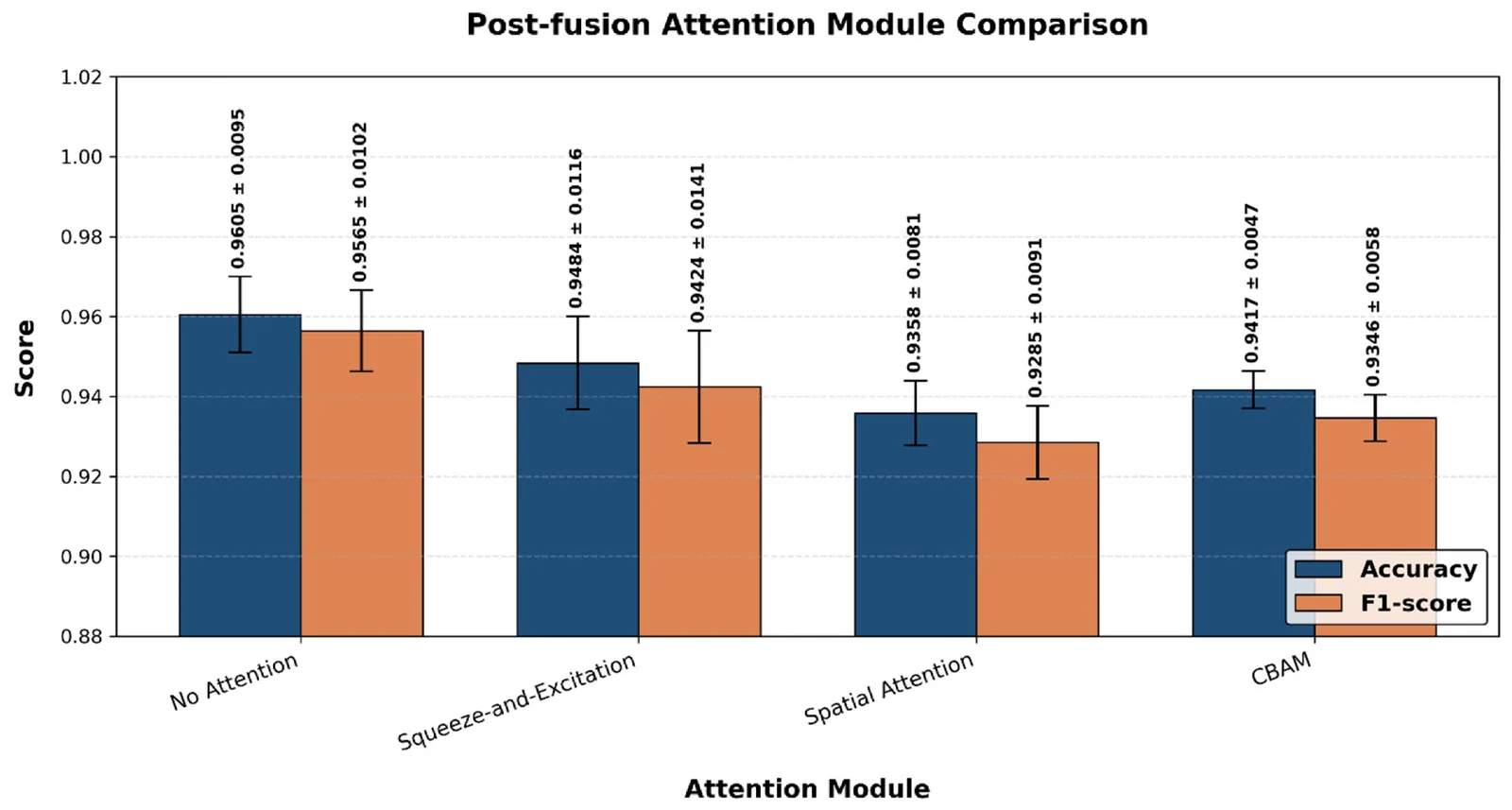
Effect of post-fusion attention on image-level accuracy and F1-score (mean ± standard deviation over three seeds).

Attention mechanisms are commonly used in DL models to refine feature representations by adaptively emphasizing informative channels or spatial regions while suppressing less relevant responses. Therefore, the final ablation study investigated whether adding an attention module after the fusion stage could further refine the fused multi-sequence representation and improve classification performance. For this purpose, three post-fusion attention mechanisms, namely squeeze-and-excitation (SE) [52], spatial attention, and the convolutional block attention module (CBAM) [65], were evaluated within the three-sequence gated-fusion DenseNet-121 model established in the previous experiments. SE performs channel-wise recalibration of feature responses, whereas the spatial attention module, corresponding to the spatial-attention submodule of CBAM, emphasizes informative spatial locations. CBAM sequentially applies channel and spatial attention within a unified attention block. Each attention module was inserted between the post-fusion refinement block and the classification head, allowing it to operate on the refined fused representation before classification. The attention-based variants were compared with the no-attention baseline, corresponding to the gated-fusion model without any additional post-fusion attention module. As in the fusion-strategy experiment, each configuration was trained using three random seeds. The image-level accuracy and F1-score values on the hold-out evaluation set are reported as mean ± standard deviation in Figure 9.

None of the evaluated attention variants improved upon the no-attention baseline. The baseline gated-fusion model achieved the highest performance, with 96.05 ± 0.95% accuracy and 95.65 ± 1.02% F1-score. Among the attention-based variants, SE was the closest to the baseline, obtaining 94.84 ± 1.16% accuracy and 94.24 ± 1.41% F1-score. CBAM achieved 94.17 ± 0.47% accuracy and 93.46 ± 0.58% F1-score, whereas spatial attention showed the lowest performance, with 93.58 ± 0.81% accuracy and 92.85 ± 0.91% F1-score.

These findings indicate that adding a dedicated attention module after gated fusion did not provide an additional benefit in this experimental setting. The gated fusion mechanism already performs adaptive, input-dependent modulation of the sequence-specific representations before aggregation; therefore, an additional post-fusion attention block may have introduced extra representational and optimization complexity without providing a corresponding gain in discriminative information. Based on these results, no post-fusion attention module was included in the final DenseGated-MSNet architecture. Taken together, the systematic ablation studies justified the final DenseGated-MSNet configuration: DenseNet-121 was selected as the backbone architecture; FLAIR sagittal, FLAIR axial, and T2 coronal were selected as the three complementary input sequences; gated fusion was adopted as the feature-level integration mechanism; and no additional post-fusion attention module was used.

## 5. Discussion

In this study, we proposed DenseGated-MSNet, a novel end-to-end DL model developed specifically for automated MS versus non-MS classification from multi-sequence brain MRI. In addition to the proposed model, we introduced a new multi-sequence brain MRI dataset for MS classification, developed to help address the limited availability of publicly accessible multi-sequence resources in this field and made publicly available to support reproducibility and future comparative studies. DenseGated-MSNet processes co-acquired MRI sequences in parallel through independent, sequence-specific DenseNet-121 branches and integrates the resulting representations using an input-dependent gated feature-level fusion mechanism. This mechanism adaptively modulates the contribution of each sequence before the final classification decision, allowing the model to exploit complementary sequence-specific information within a unified framework.

The final DenseGated-MSNet architecture was not finalized a priori, but was determined through a comprehensive series of systematic ablation studies. These analyses examined the effects of backbone architecture, individual MRI sequences and their combinations, feature-level fusion strategy, and post-fusion attention-based refinement on classification performance. The performance of the selected model configuration was internally estimated using five-fold subject-independent cross-validation, with results reported at both the image and subject levels. The hold-out results obtained during model development are presented only for completeness. Under the strict subject-independent cross-validation protocol, DenseGated-MSNet provided stable discrimination between MS and non-MS cases, with subject-level performance exceeding image-level performance. These findings position DenseGated-MSNet as a potential MRI-based research and clinical decision-support tool that complements expert clinical and radiological assessment, rather than as an autonomous diagnostic system. Given its single-center development and the absence of external validation, the model cannot yet be considered validated for general clinical diagnosis. Moreover, because the study focused on distinguishing patients with established MS from selected non-MS controls without demyelinating lesions, rather than from conditions that mimic MS, such as neuromyelitis optica spectrum disorder (NMOSD) and myelin oligodendrocyte glycoprotein antibody-associated disease (MOGAD) [66], the findings do not establish its utility for the differential diagnosis of MS in an unselected clinical population.

The superiority of the three-sequence configuration highlights the importance of exploiting complementary MRI information for automated MS classification. Although each individual sequence carried discriminative information, their diagnostic value was not equivalent, and no single sequence fully captured the heterogeneous appearance of MS-related abnormalities. The selected combination of FLAIR sagittal, FLAIR axial, and T2 coronal spans two contrasts and three acquisition planes, and may therefore reflect complementary contrast- and plane-dependent information. This finding is consistent with the radiological understanding that MS lesions and related white-matter abnormalities are emphasized differently depending on sequence contrast and acquisition plane [12,13,15,21]. Nevertheless, this complementarity was not fully predictable from acquisition geometry or from individual-sequence performance: some pairings of individually strong sequences did not improve, and occasionally slightly reduced, performance. This indicates that sequence complementarity should be established empirically rather than assumed, which is precisely what the ablation-driven selection of the final three-sequence configuration provided.

Beyond the choice of input sequences, the fusion analysis further demonstrated that the way in which sequence-specific representations are integrated is itself an important determinant of multi-sequence model performance. In the ablation experiments, gated fusion achieved the highest mean performance among the compared fusion strategies, although its advantage over channel concatenation was small and the two strategies should be regarded as broadly comparable on this dataset. In contrast, learnable weighted summation performed clearly worse, suggesting that a fixed, input-independent weighting scheme may be insufficient to capture the variable contribution of different MRI sequences across subjects and images. We therefore interpret the value of gated fusion not as a categorically superior fusion operator, but as an adaptive integration mechanism that performed at least as well as the alternatives while explicitly modeling the contribution of each sequence.

Finally, the backbone and attention-refinement analyses provide additional insight into the design of DenseGated-MSNet. DenseNet-121 provided the strongest and most consistent performance among the evaluated backbones, suggesting that a densely connected convolutional architecture was well suited to the limited-data and train-from-scratch setting of this study. In contrast, the transformer-based Swin-Tiny model performed less effectively, likely reflecting the greater data requirements of transformer architectures when trained without pretraining. Moreover, adding post-fusion attention modules did not improve performance beyond gated fusion. This suggests that once sequence-specific features had already been adaptively modulated during fusion, further attention-based refinement mainly introduced additional parameters and optimization complexity without a corresponding gain in discriminative information. Accordingly, the final DenseGated-MSNet comprised DenseNet-121, the three complementary sequences FLAIR sagittal, FLAIR axial, and T2 coronal, gated feature-level fusion, and no post-fusion attention module.

### 5.1. Comparison with Existing MRI-Based Studies

Automated MS detection and classification have been investigated using a wide range of data modalities. In addition to MRI, previous studies have explored non-imaging or alternative biomedical signals, including speech recordings [36], surface-enhanced Raman spectroscopy [37], blood-based metabolomics [41], retinal imaging [42], and gait or kinematic analysis [38,46]. Nevertheless, from a clinical and neuroradiological perspective, MRI is the cornerstone imaging modality for the diagnosis and monitoring of MS because it provides essential information on lesion distribution, temporal evolution, and disease activity [13,15]. Accordingly, MRI continues to represent the dominant imaging basis for automated MS classification studies, including the present work. Even among MRI-based studies, however, direct comparison remains challenging because the task definitions, input sequences, datasets, acquisition protocols, sample sizes, and validation protocols vary substantially. Some studies addressed binary MS versus control classification [27,28,32,39,40], whereas others considered multi-class classification involving additional neurological or demyelinating conditions such as myelitis [28,30], chronic ischemia or Alzheimer’s disease [29], or focused on lesion-level [31] or disease-activity classification [26]. Therefore, to ensure a fair and task-consistent comparison, we focused exclusively on MRI-based studies addressing the same binary MS versus control classification task as the present study.

Table 5 summarizes the comparison between the proposed DenseGated-MSNet and MRI-based studies addressing binary MS versus control classification. However, the reported performance values cannot be interpreted independently of the evaluation protocol, because differences in data partitioning directly affect the reliability and comparability of classification results. Among the directly comparable binary MS versus control studies, subject-independent evaluation is either not explicitly ensured or not clearly stated, with performance reported using image-level cross-validation [32] or hold-out validation [27,28,39,40]. To our knowledge, the present work is the only study in this directly comparable group to adopt a strictly subject-independent evaluation protocol. Image-level partitioning may introduce subject-dependent bias, because images from the same individual can appear in both training and validation sets. In this case, the model may partially learn subject-specific characteristics rather than disease-related features, leading to overly optimistic performance estimates [43,44]. To address this limitation, all experiments in the present study, including the final evaluation of DenseGated-MSNet, were conducted under a strictly subject-independent evaluation protocol, with detailed image-level and subject-level results reported in Table 5. To illustrate the impact of this methodological choice, the same model and dataset were additionally evaluated under a subject-dependent image-level hold-out setting. Under this setting, image-level accuracy increased to 99.04 ± 0.67%, compared with 96.05 ± 0.95% under the subject-independent hold-out evaluation. Notably, this subject-dependent result was comparable to the highest accuracies reported in previous binary MS versus control studies [27,28,32,39,40], suggesting that apparent performance advantages in the literature may partly reflect evaluation protocols in which subject-level separation is not explicitly enforced. Therefore, the subject-dependent result is reported only to illustrate the effect of partitioning strategy and is not considered the primary claimed performance of the proposed model.

Beyond differences in evaluation protocol, a further factor limiting direct comparison is the multi-input nature of the proposed model. DenseGated-MSNet requires a set of co-acquired MRI sequences matched at the subject level, a data structure that most existing MS classification datasets do not provide. Many available datasets rely on a single MRI sequence, and even those that include multiple sequences or modalities are not necessarily organized as matched multi-sequence inputs for the same subject. Consequently, a direct head-to-head evaluation on a shared benchmark was not feasible, and the comparison in Table 5 is necessarily based on values reported in the respective publications rather than on re-implementation under a common protocol. This limitation reflects the scarcity of publicly available multi-sequence MRI datasets specifically designed for subject-wise MS versus non-MS classification, which continues to hinder the development and fair comparison of multi-sequence models.

This gap directly motivated one of the central contributions of this work, namely the collection and public release of a multi-sequence brain MRI dataset intended to support the development and evaluation of multi-sequence MS classification models under a consistent subject-independent protocol. Together with this dataset contribution, DenseGated-MSNet is distinguished from most prior binary MS classification studies by three methodological elements: multi-sequence input processed through parallel sequence-specific branches, input-dependent gated feature-level fusion, and consistently subject-independent evaluation. To the best of our knowledge, this is among the first studies to bring these elements together for binary MS versus non-MS classification. Rather than supporting a direct claim of superiority over existing methods, which would not be fully justified given the heterogeneity of datasets and evaluation protocols, these distinctions position DenseGated-MSNet as a complementary contribution to MRI-based MS classification.

### 5.2. Sequence-Level Gate Analysis of the Gated Fusion Mechanism

Interpretability is increasingly regarded as an important requirement for DL models intended for clinical decision support, where understanding the basis of a prediction can be as important as the prediction itself. This requirement is particularly critical in multi-sequence MRI evaluation of MS, where different MRI sequences and acquisition planes offer non-equivalent diagnostic value depending on the anatomical compartment and underlying pathological tissue changes. A limitation of many multi-sequence fusion schemes is that the way in which individual inputs are combined remains largely opaque, providing limited insight into their relative contributions. In contrast, the proposed gated fusion mechanism of DenseGated-MSNet provides explicit sequence-level modulation information. Specifically, each MRI sequence is modulated by a single scalar gate, $g_{s}\;\in \;\left(0,1\right)$, before the sequence-specific features are fused. The resulting gate value therefore provides a compact indication of how strongly each sequence is modulated during fusion for a given input.

**Figure 10:**
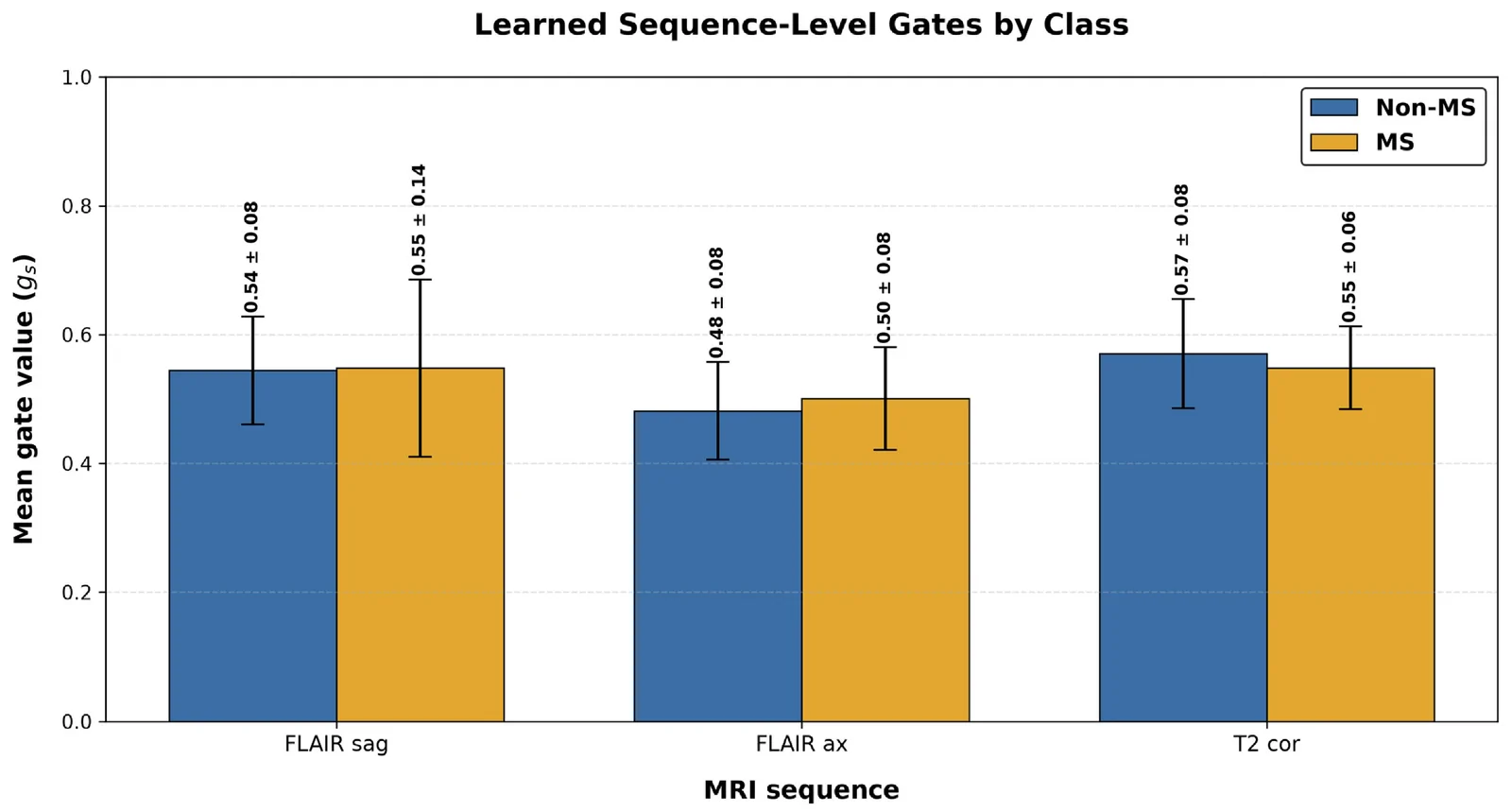
Mean learned sequence-level gate values $g_{s}$ of the final DenseGated-MSNet across the five subject-independent cross-validation folds (mean ± standard deviation).

To characterize this behavior, we extracted the gate values produced by the final DenseGated-MSNet model for each test image across the five subject-independent cross-validation folds and analyzed their distributions both overall and separately by class. In interpreting these values, the relative ordering of the gates across sequences was considered more informative than their absolute magnitude. The averaged sequence-level gate values are shown in Figure 10. Across all test images, the mean gate values were highest for T2 coronal, followed by FLAIR sagittal and FLAIR axial, with values of 0.56, 0.55, and 0.49, respectively. FLAIR axial received the lowest gate value in both non-MS controls and MS subjects, whereas T2 coronal and FLAIR sagittal received comparable values in MS subjects, indicating that the learned gating pattern was not driven by a single class but reflected a broadly consistent sequence-level modulation strategy.

According to these results, all three gates remained within a moderate range, and none approached zero, suggesting that DenseGated-MSNet did not completely suppress any input sequence during fusion. This finding supports the complementary value of the selected three-sequence configuration. These gate values should be regarded as sequence-level modulation information rather than as a direct measure of radiological importance or causal sequence contribution. To further investigate the contribution of sample-wise gate variation, the dynamic gate values were replaced with sequence-specific mean values computed from the training set of each cross-validation fold. The trained models were then re-evaluated without retraining. This modification resulted in relatively modest changes in performance, with image-level accuracy differing by at most approximately 0.7 percentage points across folds, pooled subject-level accuracy decreasing from 90.57% to 89.81%, and pooled subject-level AUC decreasing slightly from 0.947 to 0.946. These findings suggest that sample-wise variation in gate values had a limited influence on the final predictions.

Overall, the learned ordering appears to reflect complementarity among sequences rather than individual discriminative strength alone. This is consistent with the multi-sequence ablation experiments in Section 4.3.2, where combining the two FLAIR sequences yielded only a marginal improvement, whereas combining FLAIR axial with T2 coronal produced a larger performance gain. Therefore, the gate analysis suggests that the proposed fusion mechanism tends to assign greater modulation strength to sequences that add complementary information. The lower gate value of FLAIR axial is also consistent with its weaker individual performance in the single-sequence analysis reported in Section 4.3.1.

When the gates were stratified by class, the per-sequence values were very similar for non-MS controls and MS subjects, with only small differences between the two classes. The small class-wise differences indicate that DenseGated-MSNet used a broadly similar sequence-level modulation pattern for non-MS controls and MS subjects, rather than relying on markedly different MRI sequences for the two classes. Finally, the gate values should be interpreted as a coarse sequence-level description of the fusion process. They show which MRI sequences are emphasized during fusion, but they do not identify the specific image regions that contribute to the final decision. Therefore, more detailed spatial interpretability analyses, such as per-sequence saliency mapping, should be investigated in future work.

### 5.3. Limitations and Future Directions

The present study introduced DenseGated-MSNet together with a multi-sequence brain MRI dataset for MS versus non-MS classification. The proposed framework achieved competitive performance compared with existing MRI-based binary MS classification studies, while being evaluated transparently at both image and subject levels under a strict subject-independent protocol. Nevertheless, despite these promising results and methodological contributions, the present study has several limitations that should be acknowledged, as outlined below:

- The introduced dataset was collected retrospectively from a single center. Although subject-independent evaluation reduces the risk of data leakage and optimistic bias, single-center data cannot fully capture variability related to different scanners, acquisition protocols, institutions, and patient populations. Therefore, the reported performance may not directly translate to external clinical settings without further validation.
- The hold-out set used for model configuration selection also constituted one of the cross-validation folds, limiting the independence of model selection and performance estimation. Although the remaining four folds yielded comparable results, nested cross-validation or independent external validation would provide a more rigorous assessment of generalization performance.
- The multi-sequence inputs were matched at the subject level but were not spatially co-registered. Thus, different MRI sequences from the same subject were combined according to slice order rather than voxel-wise anatomical correspondence. In addition, the slice-harmonization procedure introduced a limited number of duplicated slices for sequences shorter than the subject-specific reference. These duplicated slices were also present during training; therefore, the evaluation-time sensitivity analysis cannot exclude the influence of duplication on model development. These factors may limit the extent to which the model can exploit precise cross-sequence spatial relationships.
- Post-contrast T1-weighted imaging was not systematically available and was not included as a model input; therefore, the model did not assess gadolinium-enhancing lesion activity. In addition, although spinal cord MRI findings were incorporated into the clinical reference standard where available, the model operated exclusively on brain MRI. These limitations may particularly affect patients with active enhancing disease, low brain lesion burden, or spinal-predominant involvement.
- The present study was restricted to binary MS versus non-MS classification. The proposed framework was not evaluated in more challenging differential diagnostic settings, such as distinguishing MS from other neurological or demyelinating disorders, nor in finer-grained tasks such as lesion-level classification or disease-activity prediction.
- The analysis of the fusion mechanism was limited to sequence-level gate values, which reflect sequence-level modulation rather than causal or radiological importance. Although these gates indicate which MRI sequences are emphasized during fusion, they do not identify the specific image regions that contribute to the final decision. More detailed spatial interpretability analyses, such as per-sequence saliency mapping, are therefore needed.
- The dataset remains limited in size and shows mild class imbalance. Although five-fold subject-independent cross-validation provided a more robust internal estimate of performance, the fold-to-fold variability of the subject-level results and the width of the 95% confidence intervals indicate that these estimates remain subject to non-negligible uncertainty.

Accordingly, future work should focus on addressing these limitations and further strengthening the clinical generalizability of DenseGated-MSNet. The most important next step is external validation using larger, multi-center, and ideally prospective datasets acquired across heterogeneous scanners and protocols. Methodologically, incorporating spatial co-registration and extending the framework toward three-dimensional or hybrid two-/three-dimensional modeling may allow the model to better exploit volumetric and cross-sequence anatomical information while reducing the need for slice duplication. Future studies should also extend the task beyond binary classification toward multi-class differential diagnosis (e.g., NMOSD, MOGAD [66], and cerebral small vessel disease [67]), lesion-level analysis, and disease-activity assessment. Incorporating post-contrast T1-weighted imaging to assess active neuroinflammation, together with spinal cord MRI to capture spinal involvement, would further broaden the clinical applicability of the framework. In addition, brain-masked or lesion-guided training, together with rigorous explainable-AI techniques, could help determine whether the model relies on clinically meaningful regions. Finally, cost-sensitive learning or decision-threshold optimization may be useful to reduce false-negative predictions, which is particularly important in clinical settings where missed MS cases may have serious consequences. Addressing these directions would help determine whether the subject-independent performance observed in this study can be translated into reliable performance in real-world clinical workflows.

## 6. Conclusions

In this study, we proposed DenseGated-MSNet, a novel end-to-end DL framework for MS versus non-MS classification from multi-sequence brain MRI. The model processes each MRI sequence through an independent sequence-specific branch and integrates the resulting representations using an input-dependent gated feature-level fusion mechanism. The final architecture, consisting of a DenseNet-121 backbone, a three-sequence input configuration including FLAIR sagittal, FLAIR axial, and T2 coronal images, and a gated fusion mechanism, was determined through systematic ablation studies.

In internal five-fold subject-independent cross-validation, DenseGated-MSNet achieved a pooled subject-level accuracy of 90.57% (95% CI: 86.44–93.53%) and an AUC of 0.947 (95% CI: 0.918–0.976) in discriminating established MS cases from selected non-MS controls. The learned gate values also provided sequence-level modulation information, indicating how strongly each MRI sequence was weighted during multi-sequence integration. Beyond the model itself, this study introduced a new multi-sequence brain MRI dataset to help address the scarcity of subject-wise organized resources for MS classification and to support reproducible comparisons in future research. Overall, DenseGated-MSNet provides a promising MRI-based decision-support framework that combines multi-sequence feature extraction, adaptive gated fusion, and subject-independent evaluation. Future external validation on larger and multi-center cohorts, including patients with MS mimics, is required to establish its generalizability and clinical applicability.

## Figures and Tables

**Table 1:** Summary of the state-of-the-art studies on MS classification and detection.

Ref, Year	Classes	Modality	Dataset	Method	Evaluation	Performance (%)			
						Acc	Sen	F1	AUC
[26], 2025	Active/Inactive MS lesions	MRI images (FLAIR)	3410 lesions (1408 active, 2002 inactive) from 106 patients	Custom CNN (5 convolution layers)	5-fold SICV	90.15	77.18	-	94.67
[36], 2025	MS, Healthy, OND	Speech/voice recordings	95 healthy, 99 with MS, 96 with OND	CNN + RNN (LSTM + GRU)	Hold-Out (80:20)	96.55	96.49	96.56	-
[27], 2025	Control-Ax., Control-Sag., MS-Ax., MS-Sag.	MRI images	3427 images from 60 MS patients	Lesion segmentation and RM-LSTM: recurrent MobileNet + LSTM	Hold-Out (75:25)	95.4	95.3	-	-
[37], 2025	MS, Non-MS	SERS data	96 MS and 96 control	1D-CNN	Hold-Out (80:20)	100	100	-	-
[11], 2025	MS, Non-MS (Other demyelinating diseases)	MRI scans (3D FLAIR)	CHU FBM: 182 scans (91 MS, 91 Non-MS); CHU SS: 91 MS scans; MSSEG-2: 80 MS scans	DemyeliNeXt (Few-shot prototypical network + 3D DenseNet-121 + Deep SHAP)	Few-shot evaluation	87.8	85.0	87.0	-
[38], 2025	MS, Healthy	Gait kinematic signals	19 MS, 19 healthy	Elastic Net regression	5-fold CV	-	-	-	95.0
[39], 2025	MS, Healthy	MRI images (T2-weighted)	262 MS, 163 Normal	Multi-view ResNet50 + VSAB + VCAB + QRIME + KNN	Hold-Out (80:20)	98.29	99.27	97.85	-
[28], 2025	MS, Healthy	MRI images (FLAIR)	1411 images from 72 MS, 2016 images from 59 healthy controls	**MS-Trust:** transformer + dual attention	Hold-Out (80:15:5)	92.85	91.82	92.13	98.85
[28], 2025	MS, Myelitis, Healthy	MRI images	706 images from 128 MS, 667 images from 131 Myelitis, 1373 images from 150 healthy controls	**MS-Trust:** transformer + dual attention	Hold-Out (80:15:5)	90.79	89.35	86.96	97.80
[40], 2025	MS, Healthy	MRI images (T1-w + T2-w + FLAIR)	ISBI: 82 scans; MICCAI: 45 scans	Lesion segmentation using dual patch-wise CNN + CNN for MS prediction	Hold-Out (80:20)	91.0	77.0	80.5	-
[29], 2025	AD, Chronic Ischemia, MS, Healthy	MRI images (FLAIR)	4248 images from 2100 participants	BrainNeXt	Hold-Out (70:30)	94.21	91.01	91.52	-
[41], 2024	MS, Healthy	Blood-based metabolomics data	515 MS, 241 healthy	MLP	Hold-Out (70:15:15)	87.0	82.50	-	-
[30], 2024	MS, Myelitis, Healthy	MRI images	706 images from 128 MS, 667 images from 131 Myelitis, 1373 images from 150 healthy controls	MSNet (DenseNet201 + ResNet50), FE, FS, kNN/SVM + iterative majority voting	10-fold CV	97.63	97.22	97.23	-
[42], 2024	MS, Healthy	SLO retinal vessel images	265 images from 39 MS and 106 healthy controls	Pretrained MobileNetV2 + fine-tuning	Hold-Out (repeated 5 times)	97.44	91.67	95.65	95.83
[31], 2022	With MS lesion, Without MS lesion	MRI images (FLAIR)	1544 slices from 30 MS patients; (971 with lesion, 573 without lesion)	Custom CNN (3 conv layers)	5-fold SICV	90.3	90.5	-	-
[32], 2022	MS, Healthy	MRI images (FLAIR)	1411 images from 72 MS, 2016 images from 59 healthy controls	FE + iterative NCA + KNN	10-fold CV	98.37	96.46	97.89	-

Acc: accuracy; AD: Alzheimer’s disease; Ax: axial; AUC: area under the ROC; CHU FBM: CHU Fattouma Bourguiba Monastir; CV: cross validation; CHU SS: CHU Sahloul Sousse; CNN: convolutional neural network; FE: Feature Extraction; FLAIR: fluid-attenuated inversion recovery; FS: feature selection; GRU: gated recurrent unit; KNN: k-nearest neighbors; LSTM: long short-term memory; MLP: multi-layer perceptron; MS: multiple sclerosis; MSSEG-2: MICCAI 2021 MS Segmentation Challenge; MRI: magnetic resonance imaging; NCA: neighborhood component analysis; OND: other neurological disorders; QRIME: quantum RIME; RNN: recurrent neural network; Sag: sagittal; SERS: surface-enhanced Raman spectroscopy; SICV: subject-independent cross validation; SLO: scanning laser ophthalmoscopy; SVM: support vector machine; T1-w: T1-weighted; T2-w: T2-weighted; VCAB: view channel attention block; VSAB: view space attention block.

**Table 2:** Demographic characteristics of the final cohort.

Characteristic	MS (*n* = 123)	Non-MS (*n* = 142)	*p*-Value
Age (years), mean ± SD	37.2 ± 9.1	36.9 ± 10.1	0.799
Age range (min–max)	18–61	19–63	-
Sex (Female/Male)	85/38 (69.1%/30.9%)	88/54 (62.0%/38.0%)	0.224

**Table 3:** Summary of the main hyperparameters used in this study.

Hyperparameter	Value
Optimizer	AdamW
Learning rate	0.0001
Weight decay	0.0001
Batch size	8
Dropout rate	0.4
Loss function	Cross-entropy
Maximum epochs	30

**Table 4:** Performance of the proposed DenseGated-MSNet under the subject-independent hold-out and five-fold cross-validation protocols, at the image and subject levels (mean ± standard deviation), together with the pooled out-of-fold estimates and their 95% confidence intervals (CI) for the subject-level cross-validation results.

Evaluation	Level	Performance Metrics (%)				
		Acc	Sen	Spe	Pre	F1
Hold-out	Image	96.05 ± 0.95	95.65 ± 3.44	96.39 ± 3.08	95.80 ± 3.48	95.65 ± 1.02
	Subject	97.53 ± 1.07	97.33 ± 2.31	97.70 ± 1.99	97.39 ± 2.26	97.33 ± 1.15
5-fold CV	Image	88.93 ± 2.31	87.32 ± 4.71	90.30 ± 4.61	88.66 ± 5.00	87.84 ± 2.42
	Subject	90.54 ± 4.69	89.40 ± 3.70	91.53 ± 6.96	90.55 ± 7.54	89.87 ± 4.77
	Subject, pooled (95% CI)	90.57 86.44–93.53	89.43 82.75–93.72	91.55 85.81–95.10	90.16 83.59–94.28	89.80 85.71–93.44

**Table 5:** Comparison of the proposed DenseGated-MSNet with state-of-the-art MRI-based MS versus control classification studies.

Ref, Year	Modality	Dataset	Method	Evaluation	Evaluation Level	Performance (%)			
						Acc	Sen	F1	AUC
[27], 2025	MRI images	3427 images from 60 MS patients	Lesion segmentation and RM-LSTM: recurrent MobileNet + LSTM	Hold-Out (75:25)	Image-wise	95.4	95.3	-	-
[39], 2025	MRI images (T2-w)	262 MS, 163 Normal	Multi-view ResNet50 + VSAB + VCAB + QRIME + KNN	Hold-Out (80:20)	Image-wise	98.29	99.27	97.85	-
[28], 2025	MRI images (FLAIR)	1411 images from 72 MS, 2016 images from 59 healthy controls	MS-Trust: transformer + dual attention	Hold-Out (80:15:5)	Image-wise	92.85	91.82	92.13	98.85
[40], 2025	MRI images (T1-w + T2-w + FLAIR)	ISBI: 82 scans; MICCAI: 45 scans	Lesion segmentation using dual patch-wise CNN + CNN for MS prediction	Hold-Out (80:20)	Image-wise	91.0	77.0	80.5	-
[32], 2022	MRI images (FLAIR)	1411 images from 72 MS, 2016 images from 59 healthy controls	FE + iterative NCA + KNN	10-fold CV	Image-wise	98.37	96.46	97.89	-
This study	MRI images (FLAIR Sag. + FLAIR Ax. + T2 Cor.)	3658 images per sequence from 265 subjects (123 MS, 142 non-MS controls)	DenseGated-MSNet	SI Hold Out (80:20)	Image-wise	96.05	95.65	95.65	99.29
					Subject-wise	97.53	97.33	97.33	99.86
				5-fold SICV (pooled)	Image-wise	88.93	87.33	87.83	94.23
					Subject-wise	90.57	89.43	89.80	94.72
				SuD Hold-Out (80:20)	Image-wise	99.04	97.91	98.94	99.93

Acc: accuracy; Ax: axial; AUC: area under the ROC; Cor: coronal; CV: cross validation; CNN: convolutional neural network; FE: Feature Extraction; FLAIR: fluid-attenuated inversion recovery; KNN: k-nearest neighbors; LSTM: long short-term memory; MS: multiple sclerosis; MRI: magnetic resonance imaging; NCA: neighborhood component analysis; Sag: sagittal; SuD: subject-dependent; SI: subject-independent; SICV: subject-independent cross validation; T1-w: T1-weighted; T2-w: T2-weighted; VCAB: view channel attention block; VSAB: view space attention block.

## Data Availability

The multi-sequence brain MRI dataset collected and analyzed in this study is publicly available at https://kaggle.com/datasets/37bcaead080de3ff5512c905bfea9da0f7d83b4548d0b55d2812695b906efd9a (accessed on 25 October 2026).

## Abbreviations

The following abbreviations are used in this manuscript:

1D-CNN	One-Dimensional Convolutional Neural Network
2D	Two-Dimensional
3D	Three-Dimensional
Acc	Accuracy
AD	Alzheimer’s Disease
AI	Artificial Intelligence
AUC	Area Under the Receiver Operating Characteristic Curve
Ax.	Axial
CBAM	Convolutional Block Attention Module
CC BY	Creative Commons Attribution
CHU FBM	CHU Fattouma Bourguiba Monastir
CHU SS	CHU Sahloul Sousse
CI	Confidence Interval
CNN	Convolutional Neural Network
CNNs	Convolutional Neural Networks
Cor.	Coronal
CV	Cross-Validation
DL	Deep Learning
ESNR	European Society of Neuroradiology
F1	F1-Score
FCL	Fully Connected Layer
FCLs	Fully Connected Layers
FE	Feature Extraction
FLAIR	Fluid-Attenuated Inversion Recovery
FS	Feature Selection
GAP	Global Average Pooling
GELU	Gaussian Error Linear Unit
GF-FM	Gated Feature-Level Fusion Module
GPU	Graphics Processing Unit
GRU	Gated Recurrent Unit
ISBI	International Symposium on Biomedical Imaging
JPEG	Joint Photographic Experts Group
KNN	k-Nearest Neighbors
LSTM	Long Short-Term Memory
MAGNIMS	Magnetic Resonance Imaging in Multiple Sclerosis
MICCAI	Medical Image Computing and Computer-Assisted Intervention
ML	Machine Learning
MLP	Multi-Layer Perceptron
MOGAD	Myelin Oligodendrocyte Glycoprotein Antibody-Associated Disease
MRI	Magnetic Resonance Imaging
MS	Multiple Sclerosis
MSSEG-2	MICCAI 2021 MS Segmentation Challenge
NCA	Neighborhood Component Analysis
NMOSD	Neuromyelitis Optica Spectrum Disorder
OND	Other Neurological Disorders
PACS	Picture Archiving and Communication System
Pre	Precision
QRIME	Quantum RIME
RAM	Random Access Memory
RM-LSTM	Recurrent MobileNet–Long Short-Term Memory
RNNs	Recurrent Neural Networks
ROC	Receiver Operating Characteristic
Sag.	Sagittal
SD	Standard Deviation
SE	Squeeze-and-Excitation
Sen	Sensitivity
SERS	Surface-Enhanced Raman Spectroscopy
SI	Subject-Independent
SICV	Subject-Independent Cross-Validation
SLO	Scanning Laser Ophthalmoscopy
Spe	Specificity
SuD	Subject-Dependent
SS-FEM	Sequence-Specific Feature Extraction Module
SVM	Support Vector Machine
T	Tesla
T1-w	T1-Weighted
T2-w	T2-Weighted
VCAB	View Channel Attention Block
VSAB	View Space Attention Block
